# Localization matters: nuclear-trapped Survivin sensitizes glioblastoma cells to temozolomide by elevating cellular senescence and impairing homologous recombination

**DOI:** 10.1007/s00018-021-03864-0

**Published:** 2021-06-08

**Authors:** Thomas R. Reich, Christian Schwarzenbach, Juliana Brandstetter Vilar, Sven Unger, Fabian Mühlhäusler, Teodora Nikolova, Alicia Poplawski, H. Irem Baymaz, Petra Beli, Markus Christmann, Maja T. Tomicic

**Affiliations:** 1 Department of Toxicology, University Medical Center, Mainz, Germany; 2 Institute of Medical Biostatistics, Epidemiology and Informatics, University Medical Center, Mainz, Germany; 3 Institute of Molecular Biology (IMB), Johannes Gutenberg University, Mainz, Germany

**Keywords:** BIRC5, Nuclear export signal, Inhibitor of apoptosis (IAP), Alkylation damage, Clastogenic effects

## Abstract

To clarify whether differential compartmentalization of Survivin impacts temozolomide (TMZ)-triggered end points, we established a well-defined glioblastoma cell model in vitro (LN229 and A172) and in vivo, distinguishing between its nuclear and cytoplasmic localization. Expression of nuclear export sequence (NES)-mutated Survivin (SurvNESmut-GFP) led to impaired colony formation upon TMZ. This was not due to enhanced cell death but rather due to increased senescence. Nuclear-trapped Survivin reduced homologous recombination (HR)-mediated double-strand break (DSB) repair, as evaluated by γH2AX foci formation and qPCR-based HR assay leading to pronounced induction of chromosome aberrations. Opposite, clones, expressing free-shuttling cytoplasmic but not nuclear-trapped Survivin, could repair TMZ-induced DSBs and evaded senescence. Mass spectrometry-based interactomics revealed, however, no direct interaction of Survivin with any of the repair factors. The improved TMZ-triggered HR activity in Surv-GFP was associated with enhanced mRNA and stabilized RAD51 protein expression, opposite to diminished RAD51 expression in SurvNESmut cells. Notably, cytoplasmic Survivin could significantly compensate for the viability under RAD51 knockdown. Differential Survivin localization also resulted in distinctive TMZ-triggered transcriptional pathways, associated with senescence and chromosome instability as shown by global transcriptome analysis. Orthotopic LN229 xenografts, expressing SurvNESmut exhibited diminished growth and increased DNA damage upon TMZ, as manifested by PCNA and γH2AX foci expression, respectively, in brain tissue sections. Consequently, those mice lived longer. Although tumors of high-grade glioma patients expressed majorly nuclear Survivin, they exhibited rarely NES mutations which did not correlate with survival. Based on our in vitro and xenograft data, Survivin nuclear trapping would facilitate glioma response to TMZ.

**Supplementary Information:**

The online version contains supplementary material available at 10.1007/s00018-021-03864-0.

## Introduction

Among the different subtypes of brain cancer, glioblastomas (WHO grade IV) account for the majority (> 50%) of malignant brain tumors [1, 2]. Currently, the standard protocol for high-grade gliomas (HGG) is maximal safe resection followed by radiotherapy with concomitant or adjuvant temozolomide (TMZ) [3]. Since TMZ exerts its cytotoxic effect by the induction of O^6^-methyguanine (O^6^MeG), and subsequent formation of DNA double-strand breaks (DSBs), success of glioblastoma therapy strongly depends on the DNA repair capacity of the tumor. In the absence of O^6^-methylguanine-DNA methyltransferase (MGMT), O^6^MeG is not repaired and mispairs during DNA replication with thymine, resulting in GC > AT transition mutations. Persistent O^6^MeG can be converted into DSBs via futile DNA mismatch repair (MMR) in the second replication cycle after TMZ exposure [4]. If these DSBs are not repaired by homologous recombination (HR), they result in chromosome aberrations and the activation of cell death via apoptosis [5, 6]. Majorly, however, TMZ induces senescence in p53/p21-proficient glioma cells, an irreversible cell cycle arrest, which represents an important survival mechanism, impacting TMZ-based therapy of malignant gliomas [7]. Error-prone non-homologous end-joining (NHEJ) seems not to protect against O^6^-methyguanine – triggered DSBs, chromosome aberrations and cell death [8].

One of the most prominent tumor resistance factors and a putative target of anticancer therapy is Survivin, the smallest inhibitor of apoptosis (IAP). Survivin is overexpressed in virtually all human cancers, whereas it is only marginally found in differentiated adult cells, mainly in the G2/M phase of the cell cycle [9]. As essential member of the chromosome passenger complex (CPC), Survivin is crucially involved in the correct segregation of the chromosomes replicated in the S-phase [10] and is essential for the completion of cytokinesis [11]. In addition to the passive diffusion of the small (16.5 kDa) Survivin protein into different cell compartments, the intracellular distribution is influenced by active export processes, which are mediated via the C-terminal nuclear export sequence (NES) [12]. Without this active export, tumor-protective effects are reduced, since Survivin cannot accumulate in the cytoplasm or in the mitochondria [13]. The purpose of the study was to analyze whether TMZ, a component of the first-line glioma therapy, influences intracellular localization of Survivin. Furthermore, we addressed the question whether its differential localization alters the reproductive survival of glioblastoma cells and whether this is associated with changes in the repair of DSBs by HR, induction of chromosome aberrations and the onset of senescence. We were also keen to know whether, dependent on Survivin localization, TMZ induces different transcriptionally regulated pathways, and whether upon TMZ-induced genotoxic stress Survivin physically interacts with any of the DNA repair factors. Further, we aimed to verify our in vitro findings in an orthotopic intracranial glioblastoma xenograft model and to analyze whether nuclear accumulation of Survivin in HGG patients’ tissue is the consequence of NES mutations.

## Materials and methods

### Cell culture, plasmid and siRNA transfection, drugs, and chemicals

The human glioblastoma cell line LN229 (RRID:CVCL_0393) was purchased from LGC Standards and cell lines U87 (since misidentified, it refers to as ‘glioblastoma of unknown origin’; RRID:CVCL_0022) and A172 (RRID:CVCL_0131) were obtained from CLS cell lines service. The stable Survivin-expressing clones were generated by Effectene (Qiagen) based transfection of LN229 and A172 cells with the pcDNA3.1 plasmid carrying the cDNA of *BIRC5* as wild-type copy or as a Survivin variant (SurvivinNESmut) mutated in the nuclear export sequence (NES) [10] both tagged to eGFP. The SurvNESmut-GFP vector was verified by sequencing. The alignment with *BIRC5* wild-type sequence (NM_001168.2) shows that both inactivating mutations were found at the anticipated site within the NES (Suppl. Fig. S2a). Single G418-resistant clones were analyzed for expression and localization of the fusion protein. LN229-RAD51sh cells [5] were co-transfected with pcDNA3.1-Surv-GFP and pSV-puro and single clones selected by G418 (0.75 mg/mL) and puromycin (5 µg/mL). Cells were grown in DMEM containing 10% FBS (Invitrogen) at 37 °C, 6% CO_2_. Transfections of *small interfering RNAs* (SignalSilence® Survivin siRNA, Cell Signaling Technology #6351; Survivin siRNA (h), Santa Cruz sc-29499; RAD51 siRNA (h), sc-36361; Origene’s 27-mer RAD51 siRNA) were performed using Lipofectamine RNAiMAX Reagent (Invitrogen), as described [14]. TMZ (Sigma) was dissolved to a 35 mM stock solution in dimethyl sulfoxide (DMSO) and stored in aliquots at −80 °C. Leptomycin B (LMB; Sigma) was PBS-diluted and stored at −20 °C.

### RNA preparation and real-time qPCR

Total RNA was isolated using the NucleoSpin^®^ RNA Kit (Macherey–Nagel, Düren, Germany) and 1 µg RNA was transcribed into cDNA (Verso cDNA Kit, Thermo Scientific, Dreieich, Germany). qPCR was performed in technical triplicates using the GoTaq^®^ qPCR Master Mix Protocol (Promega, A6001/A6002, Madison, USA) and the CFX96 Real-Time PCR Detection System (Biorad, München, Germany). The specific primers are listed in Suppl. Table S1. Non-transcribed controls were included in each run, expression was normalized to *gapdh* and *β-actin*; the untreated control was set to 1. Analysis was performed using CFX Manager^™^ Software; SD shows intra-experimental variation.

### Protein extract preparation for western-blot analysis and mass-spectrometry-based interactomics

Whole-cell extract preparation and western-blot analyses were described [7]. Antibodies are specified in the Suppl. Table S2. For fractionated cellular extracts two separate buffers for nuclear and cytoplasmic extracts were used. The cell pellet was resuspended in 500 μl PBS and split in half. To isolate cytoplasmic protein fraction only, the first pellet was lysed in 200 μl lysis buffer 1 (10 mM NaCl, 3 mM MgCl_2_, 10 mM Tris–HCl (pH 7.4)) with freshly added 1 mM DTT and 1 mM PMSF on ice for 5 min. After adding 1% NP-40, suspension was incubated for 3 min and centrifuged (1 min, 10,000 rpm, 4 °C). To extract the nuclear protein fraction, the second pellet was first lysed with buffer 1 on ice for 5 min and then for 6 min with 1% NP-40. Nuclei were centrifuged (5 min, 3000 rpm, 4 °C), then resuspended in 500 μl lysis buffer 1 and directly centrifuged. The so purified nuclei were then lysed in 100 μl lysis buffer 2 (20 mM Tris–HCl, pH 7.4), 40 mM Na_4_P_2_O_7_, 5 mM MgCl_2_, 10 mM EDTA, 1% Triton-X100, 1% SDS) with freshly added 1 mM DTT and 1 mM PMSF. To ensure complete lysis of the nuclear membrane, samples were sonicated with 3 × 10 pulses at 40% duty cycle. After centrifugation of the lysate (1 min, 10,000 rpm, 4 °C), supernatant was frozen. For mass spectrometry-based interactomics, see detailed protocols in Supplementary methods.

### Determination of cell death, cellular senescence, and analysis of chromosome aberrations

Colony formation (CFA) and MTT assays were conducted as described [14, 15]. Cell cycle distribution and apoptosis acquisition were performed using flow cytometry (FACSCanto II, DIVA Software, BD Biosciences), as described [7]. Senescence was determined by β-Galactosidase (SA-β-Gal) staining [7] and analyses of chromosome aberrations were performed as described [16].

### DSB repair activity assays

HR efficiency was determined by a qPCR-based HR activity kit (Norgen Biotek Corporation, ON, Canada), and in detail described [14]. The abundance of a recombined (repaired) HR-plasmid was quantified by relative qPCR. The expression of the HR-product was calculated using an untreated sample as a calibrator control and an internal plasmid sequence as reference target. qPCR was performed in technical triplicates using the GoTaq^®^ qPCR Master Mix Protocol (Promega, Madison, USA) and the CFX96 Real-Time PCR Detection System (Biorad, München, Germany). Analysis was performed using CFX Manager^™^ Software; SD shows intra-experimental variation. NHEJ efficiency was assessed by the DNA-PKcs-dependent repair activity assay (Promega) and in detail described in the Supplementary information.

### Immunofluorescence staining of cells and FFPE tumor tissue sections

Procedures for immunofluorescence staining including foci detection of single cells and FFPE tissue sections were described previously [14, 17]. Antibodies are listed in the Suppl. Table S2. Tissues were visualized on an LSM 710 (Carl Zeiss GmbH) using an EC Plan-Neofluar 10x/0.3, or C-Apochromat 40x/1.2 W Korr M27 objective. Standard hematoxylin–eosin (HE) staining was visualized on Echo Rebel REB-01 hybrid microscope (20x/0.40 LWD Achromat Phase objective).

### Pyrosequencing and Kaplan–Meier estimates

Pyrosequencing primers identify two NES-inactivating transitions in genomic DNA at bp 278 T > C and bp 292 C > T as well as one silent transition in between (A288G) (for set-up and primer sequence, see Suppl. Fig. S10a). Kaplan–Meier estimates for the overall survival (OS) were calculated upon stratification for the *BIRC5*-NES mutation status for grade III and IV gliomas, separately. Patients with a NES-inactivating SNP > 5% at one of the respective positions were designated as potentially “SurvNESmut”, those with SNP frequency ≤ 5% as “SurvNESwt”. The survival differences in both groups were tested for statistical significance by log-rank test (Mantel-Cox test) and were computed using SPSS 23 (IBM). Investigation on anonymized patients’ material was approved by the authors' institutional interdisciplinary neurological review board. The HGG patients’ samples (collected 2010–2013) were described in detail [17].

### Transcriptional (RNA-Seq) analyses

Cell clones were left unexposed or were exposed to 50 µM TMZ for 48 h and total RNA was isolated using RNA isolation Kit (Machery-Nagel, Düren, Germany). Highly pure RNA was sequenced on the Illumina platform (StarSEQ, Mainz, Germany), and the RNA-Seq raw data were statistically re-analyzed using EdgeR and quasi-likelihood *F *test (QLF) [18, 19]. Raw sequencing data were deposited in GEO (GSE154337). Prior to Gene Onthology (GO) term analysis, the datasets were pre-filtered for *p *values. For the intra-clonal analysis of TMZ-induced gene expression changes, and the normalized inter-clonal comparison of Survivin localization on the TMZ response, we obtained few significant hits. Therefore, the threshold was increased to a p-value of 0.25. GO term analyses were carried out using DAVID 6.8 web tools (https://david.ncifcrf.gov/https://david.ncifcrf.gov) [20].

### Animal experiments

An orthotopic intracranial murine model [21] was used to evaluate the tumorigenic potential of glioblastoma cells and the derived clones with differential subcellular localization of Survivin. Experiments were conducted at the Translational Animal Research Center (TARC) of the University Medical Center Mainz with the approval by the State Office of chemical investigations of Rhineland-Palatinate, Mainz, Germany (permission #23 177-07/G19-1-014). Mice were maintained, operated, treated and terminated in accordance with the guidelines and policies for animal experimentation, housing and care, as documented in the European Convention for the Protection of Vertebrates Used for Scientific Purposes. A detailed experimental description according to the NIH ARRIVE guidelines can be found in the Suppl. Table S3. Immunodeficient (nude) mice (strain NMRI *Foxn1*^*nu/nu*^, six-week females, body weight 21–27 g) were obtained from Charles River Europe. For intracranial implantation, single cell suspensions (LN229, Surv-GFP or SurvNESmut-GFP) were washed twice in PBS and re-suspended at 15.000 cells/µL, and 3 µL were injected into the caudato-putamen of the right hemisphere using a stereotactic frame (TSE Systems, Bad Homburg, Germany) with the following coordinates: 1 mm (anteroposterior axis), 3 mm (lateromedial axis), and 2.5 mm (vertical axis), in reference to the bregma. Three weeks post-implantation, mice (*n* = 7 per group) were injected with TMZ (diluted in 10% DMSO/0.9% NaCl, 5 mg/kg body weight) five times a week for 4 weeks, and followed for 6 months after the end of the treatment. Mice were terminated at the manifestation of neurological symptoms related to brain tumor. For immunofluorescence, paraffin-embedded brains were sectioned at 1–3 µm thickness.

### Statistics

The data were evaluated using two-way analysis of variance (Two-way ANOVA) followed by Bonferroni-correction and were expressed as a mean ± SD, or, where indicated, by One-Way ANOVA with Tukey’s post hoc analysis or by Student’s *t* test. p* ≤ 0.05 was considered statistically significant, p** ≤ 0.01 very significant, p*** ≤ 0.005 highly significant and p**** ≤ 0.001 most significant. Statistical analyses were performed using GraphPad Prism version 6.01 for Windows, GraphPad Software, La Jolla California USA.

## Results

### Subcellular localization of Survivin-GFP variants impacts clonogenic glioblastoma cell survival

**Fig. 1:**
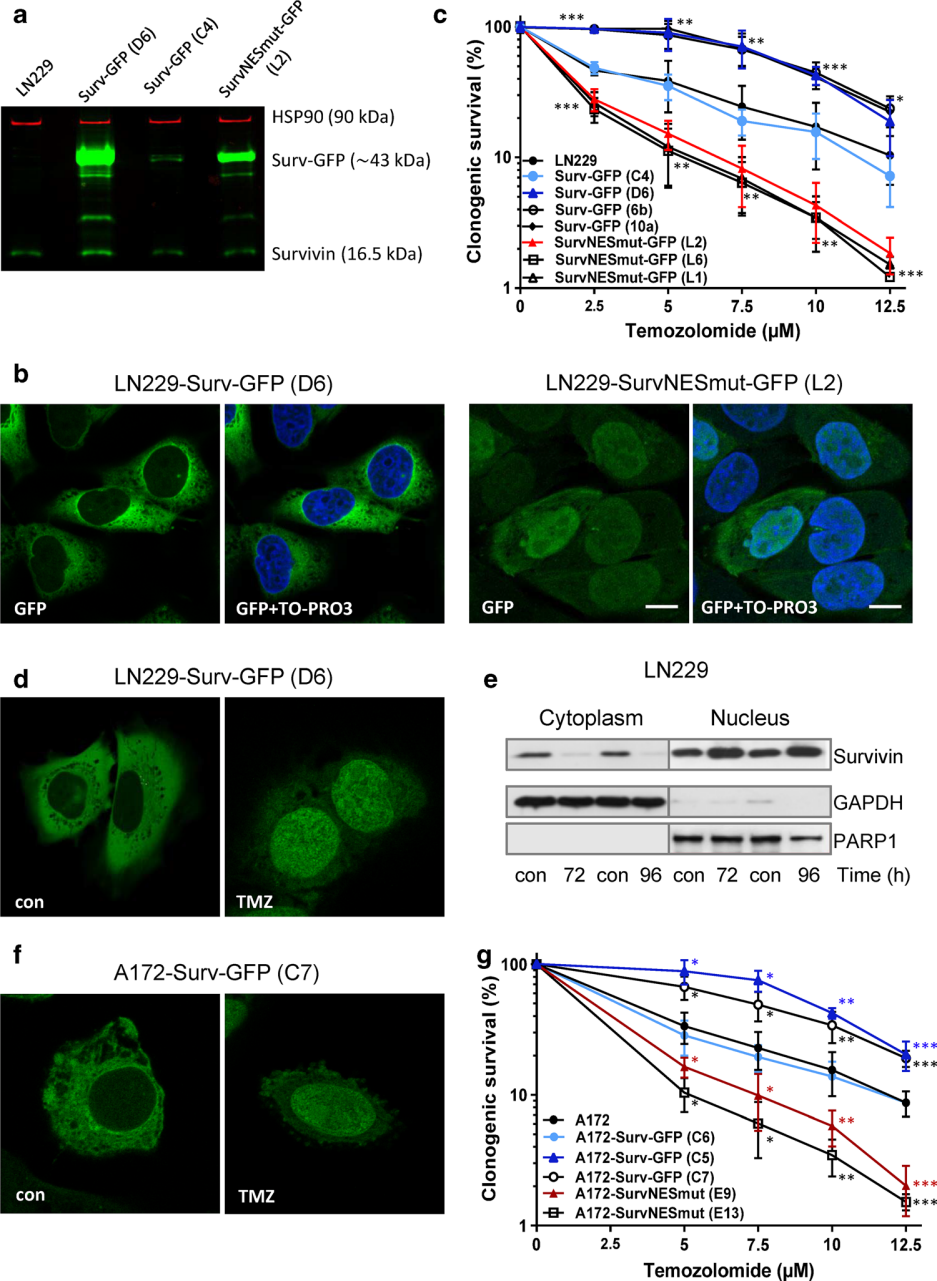
**a** Western-blot analysis of LN229 clones expressing the Survivin-GFP and mutated Survivin NESmut-GFP fusion protein. HSP90, loading control **b** Immunofluorescence staining showing localization of Survivin-GFP and Survivin NESmut-GFP protein (clones D6 and L2). Nuclei were stained with TO-PRO3 (depicted in blue); scale bars equate 10 μm. **c**, **g** colony formation of LN229, A172 and the isogenic Survivin-expressing clones upon TMZ. Three independent experiments in technical triplicates were performed ± SD. Test for statistical analysis was performed by Two-Way ANOVA with Bonferroni correction, comparing TMZ-treated clones with TMZ-treated parental cells. p* ≤ 0.05 statistically significant, p** ≤ 0.01 very significant, p*** ≤ 0.005 highly significant, p**** ≤ 0.001 most significant. **d**, **f** immunofluorescence staining showing nuclear translocation of Survivin-GFP (clone D6 and clone C7) in LN229 and A172 cells, respectively, after exposure to 50 µM TMZ. **e** Survivin expression in fractionated LN229 extracts. GAPDH and PARP1, loading controls

To investigate the impact of Survivin’s subcellular localization on the response to TMZ, we generated a panel of stable Survivin-expressing clones of the *glioblastoma multiforme* (grade IV) LN229 and A172 cell line. For a majority of the experiments we used well-defined, tumorigenic LN229 cells which do not express MGMT, have functional MMR, are p53/p21-proficient and, thus, responsive to TMZ in terms of induction of cell death and senescence [7]. Since A172 generally grow very slowly (doubling > 48 h) and do not build xenografts, we used this cell line (also MGMT-deficient, MMR-proficient and p53/p21-proficient) and the isogenic clones only for the verification of crucial in vitro results. The generated clones express either wt-Survivin fused to GFP (in further text LN229-Surv or A172-Surv), or a fusion protein mutated in the Survivin nuclear export sequence (LN229-SurvNESmut or A172-SurvNESmut), as shown by western-blot (Fig. 1a, Suppl. Fig. S1a/b). Of note, endogenous Survivin is much lower expressed as compared to the fusion protein.

The introduced NES mutations (one transversion (T > G or C > G) and one transition (T > C) in each codon) led to the substitutions of leucine by alanine at positions 96 and 98 (Suppl. Fig. S2a), not changing any other biological properties of Survivin except its localization site [12]. As expected, non-mutated Surv-GFP was predominantly localized to the cytoplasm (Fig. 1b, left panels; Suppl. Fig. S2b, upper panels). In contrast, a nuclear trapping could be observed for the cell clones carrying the NES-deficient Surv-GFP variant (Fig. 1b, right panels; Suppl. Fig. S2b, lower panels). Importantly, all LN229 and A172 clones expressing Surv-GFP showed survival advantage towards TMZ, as compared to SurvNESmut clones that formed significantly fewer colonies (Fig. 1c, g). At 10 µM TMZ, Survivin-expressing clones showed a survival rate of ∼40%, whereas in the SurvNESmut clones it dropped to ∼4%. The LN229-Surv (C4) and A172-Surv (C6) exhibited similar survival rates as the parental cells (∼20%) (for stained colonies, see Suppl. Fig. S3a). The results exhibit an essential role of Survivin compartmentalization in protection against TMZ-based chemotherapy.

### Survivin translocates to and accumulates in the nucleus upon genotoxic stress

Beside mutations in the NES, localization of Survivin can be influenced by substances that interfere with processes or components of nuclear export. Thus, the CRM1 inhibitor Leptomycin B (LMB), used as positive control, led to nuclear accumulation of Survivin-GFP (Suppl. Fig. S3b).

To analyze whether chemotherapy-relevant drugs trigger redistribution of Survivin from the cytoplasm into the nucleus LN229-Surv and A172-Surv cells were exposed to 50 µM TMZ. We observed a significant nuclear accumulation of the fusion Surv-GFP protein, visualized as pan staining, not as condensed foci (Fig. 1d, f; Suppl. Fig. S2c). In support, we also observed a strong decrease in the endogenously expressed cytoplasmic Survivin and a parallel increase in the nuclear fraction, 72 h after exposure of LN229 cells to 100 µM TMZ (Fig. 1e).

### TMZ-induced cell cycle arrest, polyploidy and senescence is more pronounced in SurvNESmut clones

**Fig. 2:**
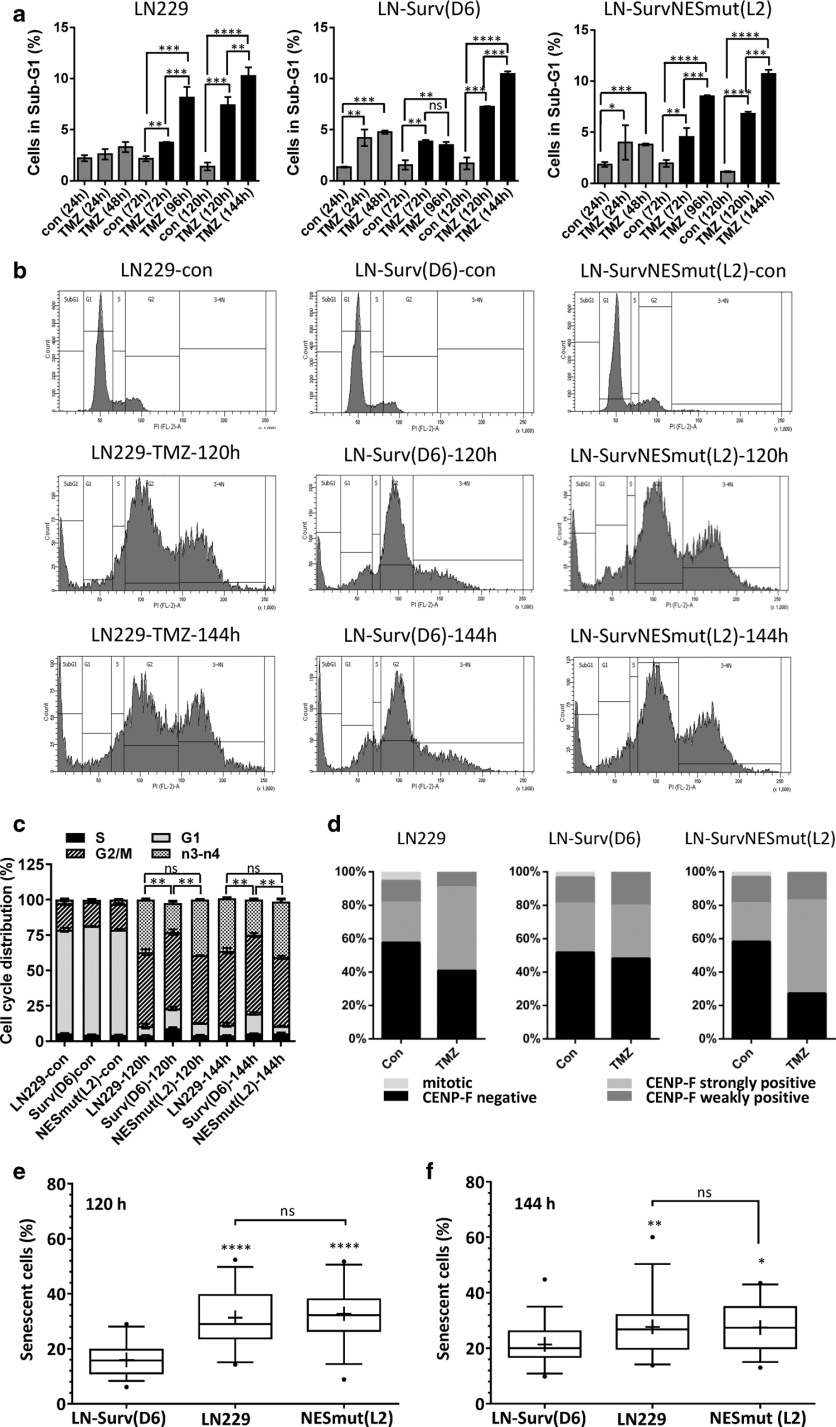
**a** Flow cytometric analysis with quantification of cell death (SubG1) and **b** corresponding histograms and **c** cell cycle distribution upon exposure of LN229 and LN229-Surv clones (D6 and NESL2) to 50 µM TMZ. Test for statistical analysis was performed by Two-Way ANOVA with Bonferroni correction. **d** CENP-F staining of unexposed and to 50 µM TMZ-exposed LN229 and the clones (D6 and NESL2) for 120 h. A representative experiment from two independent staining experiments is shown. **e/f** Box-plots showing senescence induction as fraction of SA-ß-Gal positive cells exposed to 50 μM TMZ for 120 or 140 h. For each group 250–500 cells were counted in each independent experiment (*n* = 3). Whiskers indicate 5th and 95th percentile, with boxes representing first, second (median) and third quartile (from top to bottom). Geometric means are marked with “ + ”. Outliers (values out of 5 – 95 percentile range) are marked as “•”. Test for statistical significance was performed by One-Way ANOVA with Bonferroni post hoc analysis. p* ≤ 0.05 statistically significant, p** ≤ 0.01 very significant, p*** ≤ 0.005 highly significant, p**** ≤ 0.001 most significant

Survivin is known to increase survival by reduction of cell death (apoptosis). To test whether this accounts for the increased clonogenic survival of LN229-Surv cells upon TMZ, the apoptotic cell fraction was determined. The LN229-Surv clones were protected against TMZ for up to 96 h after exposure but at later time points (120 and 144 h post-TMZ) there were no differences in SubG1 rates between LN229-Surv, LN229-SurvNESmut and LN229 (or Surv-C4) cells (Fig. 2a, Suppl. Fig. S4a). This was supported by equal frequency of early and late apoptosis using AnnexinV/PI staining (Suppl. Fig. S4b). Thus, the strong differences in clonogenic survival cannot be caused by reduction of TMZ-induced cell death alone. Based on the cell cycle distribution data (Fig. 2b, c), the LN229-Surv and LN229-SurvNESmut clones were comparably arrested in G2, however, the staining with the G2-marker CENP-F undoubtedly revealed a lower fraction of LN229-Surv cells in G2 (Fig. 2d), also exhibiting significantly less polyploid (n3–n4) cells, compared to LN229 or the SurvNESmut cells (Fig. 2b, c; Suppl. Fig. S4c). In support, upon TMZ, a strong staining with the microtubule marker α-tubulin in LN229-Surv cells implied significant mitotic entry, opposite to SurvNESmut cells (Suppl. Fig. S4d).

Recently we could demonstrate that TMZ efficiently induces senescence in p53/p21-active glioblastoma cells out of the G2-phase and highlighted the potential mechanism based on the regulation by the E2F signaling [7]. To examine whether exogenous expression of intact Survivin or SurvNESmut influences cellular senescence upon TMZ, SA-β-Gal staining was conducted. Indeed, the frequency of senescence was significantly reduced in LN229-Surv and A172-Surv cells (Fig. 2e, f, Suppl. Fig. S5a–c), in contrast to the cells expressing SurvNESmut showing enhanced senescence, which could explain CFA values. Representative pictures of the different Survivin-expressing cells undergoing senescence are shown in Suppl. Fig. S5e.

### TMZ induces more DSBs in SurvNESmut clones and upon Survivin knockdown in parental cells

**Fig. 3:**
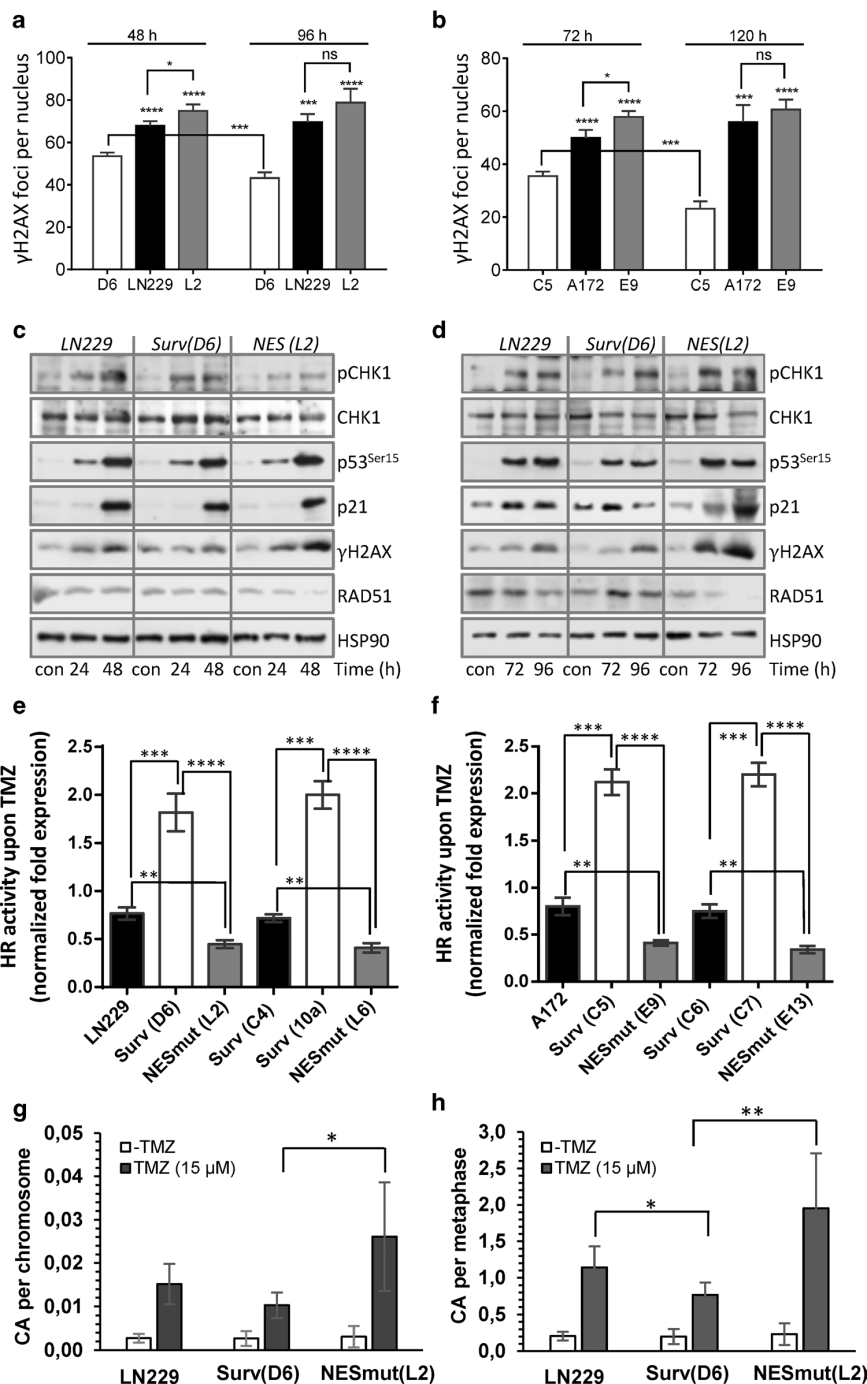
**a**, **b** Quantification of DSB induction (γH2AX foci formation per nucleus) in LN229 and A172 cells and the isogenic clones exposed to 50 μM TMZ. Error bars indicate SEM. Test for statistical significance was performed by One-Way ANOVA with Tukey’s post hoc analysis; *p *values (*) indicated above each column were calculated between LN229 Surv-GFP (clone D6) / A172 Surv-GFP (clone C5) and the corresponding column. **c**, **d** Western-blot analyses of DDR in LN229 and the clones exposed to 50 µM TMZ for 24, 48, 72 and 96 h. Representative blots from three independent experiments are shown. HSP90, loading control. **e**, **f** qPCR-based HR activity was measured in unexposed and to 50 µM TMZ-exposed LN229 and A172 derived cell clones, respectively. 72 h later, the cells were transfected with the HR plasmids and 24 h thereafter subjected to isolation of total cellular DNA. The normalized fold expression is shown, using unexposed LN229 or A172 clones as calibrator control and the internal reference plasmid sequence for normalization (ΔΔCT). The data are the mean of two independent experiments performed in technical duplicates ± SD. **g**, **h** Induction of chromosome aberrations (per chromosome and per metaphase) in unexposed LN229 and the isogenic clones, or cells exposed to 15 µM TMZ. The mean of three independent experiments is shown. p* ≤ 0.05 statistically significant, p** ≤ 0.01 very significant, p*** ≤ 0.005 highly significant, p**** ≤ 0.001 most significant

As TMZ causes cell cycle arrest in the G2-phase, the main repair pathway of TMZ-induced DSBs is HR. A supporting function of Survivin in the repair of ionizing radiation-induced DSBs by NHEJ in glioma cells has been suggested [22] and the physical and spatial interaction of Survivin with DNA-PKcs in irradiated cells recently verified [23]. To elucidate the impact of Surv-GFP and SurvNESmut-GFP on induction and repair of DSBs after TMZ treatment, γH2AX foci formation was analyzed as surrogate marker for DSBs. Distinctly visible γH2AX foci within cell nuclei of LN229, A172 and their isogenic clones (Suppl. Fig. S6a) were quantified 48 and 96 h as well as 72 and 120 h upon TMZ, respectively (Fig. 3a, b). At all times LN229 and A172 cells as well as the isogenic SurvNESmut clones and the low-expressing LN229-Surv clone C4 (Suppl. Fig. S6b) showed a higher induction of γH2AX foci compared to LN229-Surv or A172-Surv clones, pointing to a defect in either signaling or processing of TMZ-induced damage. As a proof for unrepaired DSBs, co-localization of γH2AX with the DSB marker, 53BP1 was shown in TMZ-exposed LN229 cells (Suppl. Fig. S6c). To examine whether differences in the formation of γH2AX foci were caused by different DNA damage response (DDR), the pCHK1-p53-p21 axis was analyzed in TMZ-exposed clones (Fig. 3c, d). Initial DDR signaling among the clones was unaltered (Fig. 3c), nevertheless, at later times, the expression of p21 in SurvNESmut cells was sustained (Fig. 3d). Concordant with foci induction, LN229 and LN229-Surv clones also showed lower γH2AX expression. Moreover, expression of RAD51 was nearly unaffected in these cells, while LN229-SurvNESmut exhibited increased γH2AX and a strongly reduced RAD51 expression (Fig. 3c, d), hinting at compromised repair.

**Fig. 4:**
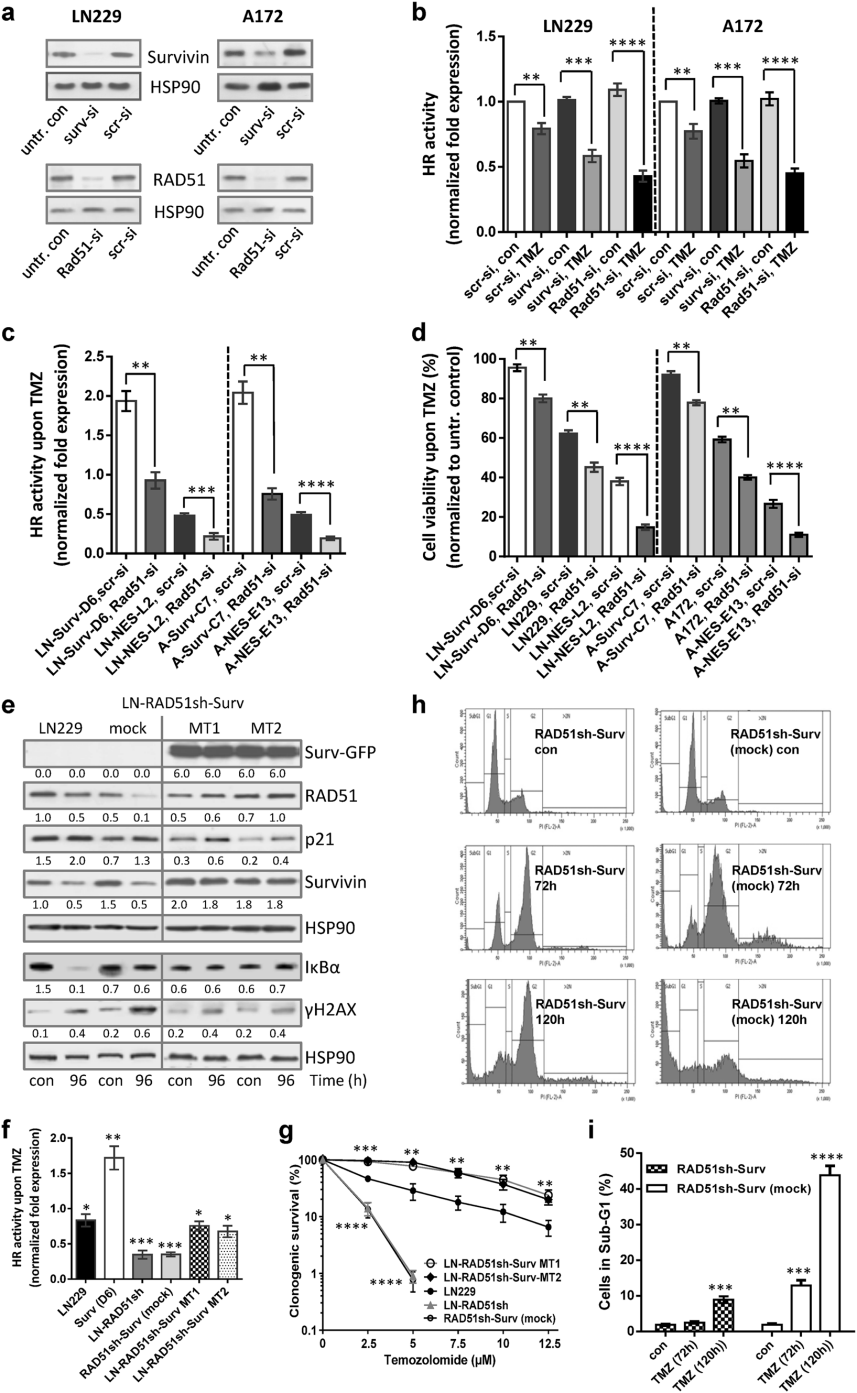
**a** Knockdown of Survivin and RAD51 in LN229 and A172 cells. HSP90, loading control. **b** Effect of Survivin and RAD51 knockdown on HR activity in unexposed and to 50 µM TMZ-exposed LN229, A172 and **c** the isogenic clones. 72 h later cells were transfected with the HR plasmids and 24 h thereafter subjected to isolation of total cellular DNA. The normalized fold expression is shown, using unexposed clones or unexposed scr-siRNA-transfected LN229 and A172 cells, respectively. Two independent experiments were performed in technical duplicates ± SD. **d** Effect of RAD51 knockdown on cell viability after exposure to 50 µM TMZ in Surv and SurvNES clones of LN229 and A172 cells, respectively. Scr-si, scrambled siRNA. **e** Western-blot analyses of DDR and repair proteins in LN229 cells, the isogenic clone with stable RAD51 knockdown but without Survivin overexpression (LN-RAD51sh-Surv mock) and the clones expressing Survivin in the RAD51kd background (LN-RAD51sh-Surv MT1 and MT2) upon exposure to 50 µM TMZ for 96 h. Representative blots from three independent experiments are shown. Densitometric analysis (fold-induction) was evaluated in relation to loading control, HSP90. **f** HR assay in different clones exposed to 50 µM TMZ for 72 h compared to untreated control set to 1. The data represent two independent experiments in technical duplicates ± SD. **g** Colony formation assay in LN229, RAD51sh, RAD51sh-Surv (MT1, MT2) and RAD51sh-Surv (mock) clones exposed to TMZ. Three independent experiments in technical triplicates were performed ± SD. **h** Cell cycle distribution of RAD51sh-Surv and RAD51sh-Surv (mock) cells unexposed or exposed to 50 µM TMZ for 72 and 120 h, and **i** quantification of dead (subG1) cells. Two independent experiments in technical duplicates were performed ± SD. **b**, **c**, **d**, **f**, **g**, **i** Test for statistical analysis was performed by two-Way ANOVA with Bonferroni correction. p** ≤ 0.01 very significant, p*** ≤ 0.005 highly significant, p**** ≤ 0.001 most significant

To clarify whether Survivin localization is associated with DNA repair capacity, we determined the HR activity in different Surv and SurvNESmut clones upon TMZ (Fig. 3e, f). In line with the lower levels of γH2AX foci in LN229-Surv and A172-Surv cells, TMZ-induced HR activity was ∼twofold higher, as compared to the untreated controls set to 1. Notably, upon TMZ, SurvNESmut clones showed a stronger impairment in HR activity in comparison to LN229 or A172 (Fig. 3e, f), which reflects the lower amount of RAD51 protein, the higher number of remaining γH2AX foci and the increase in sensitivity to TMZ. Also, the knockdown of Survivin in LN229 and A172 cells (Fig. 4a, upper panel) diminished the HR activity upon TMZ by ∼0.5-fold (Fig. 4b) uncovering putative Survivin’s role in the repair of TMZ-induced DSBs.

In line with impeded DNA repair and increase in DSBs, LN229-SurvNESmut (L2) cells exhibited significantly more TMZ-induced chromosome aberrations in comparison to LN229-Surv (D6) cells (Fig. 3g, h). Spectrum of aberration types, implying defects in HR is shown in Suppl. Table S4. Therefore, in contrast to nuclear-trapped Survivin, cytoplasmic Survivin protects glioblastoma cells against TMZ-induced clastogenic effects and thereby against senescence and cell death.

### Cytoplasmic Survivin partially rescues reduced HR activity and compensates for cell survival

To further substantiate the supportive role of Survivin in the repair of DSBs by HR, we silenced *RAD51* (Fig. 4a, lower panel) and examined HR repair efficiency of LN229 and A172 cells (Fig. 4b) and of different Surv and SurvNESmut clones (Fig. 4c) upon TMZ. As expected, upon RAD51 knockdown, the HR activity was significantly reduced in the parental cells and was even more impaired in the SurvNESmut clones (Fig. 4c). Most interestingly, despite RAD51 knockdown, the clones with cytoplasm-expressed Survivin (LN229-Surv, A172-Surv) still showed pronounced HR activity, reaching the repair efficiency of TMZ-exposed parental cells (see Fig. 3e). This might reflect the supportive role of free-shuttling cytoplasmic, not nuclear-trapped Survivin, in DNA repair. The RAD51 knockdown in LN229-Surv or A172-Surv reduced cell viability after TMZ exposure by  ~ 20%, in comparison to Surv clones without silenced RAD51 (Fig. 4d). Surprisingly, LN229-Surv-RAD51kd and A172-Surv-RAD51kd cells survived significantly better than LN229 or A172 cells with intact RAD51 and without Survivin overexpression. This, at first, unexpected result let us conduct vice versa experiment. Thus, we expressed Survivin in RAD51sh cells (stable ~ 50% knockdown; [5]) and compared protein expression, HR activity and survival in two independent LN-RAD51sh-Surv clones (MT1, MT2), RAD51sh or LN-RAD51sh-Surv (mock) and LN229 cells (Fig. 4e). Interestingly, upon TMZ, the RAD51 and Survivin expression was stabilized in the two RAD51sh-Surv clones in comparison to RAD51sh-Surv (mock) cells, where it was strongly reduced. The RAD51sh-Surv cells exhibited similar HR activity like LN229 (Fig. 4f), verifying the data on transient RAD51 knockdown. Of note, the NF-κB signaling, known to support TMZ-induced senescence [7], detected by IκBα degradation, was not activated in LN-RAD51sh-Surv and LN-RAD51sh, in contrast to LN229 cells (Fig. 4e). The colony formation (Fig. 4g) and cell cycle/SubG1 values (Fig. 4h/i) of the two independent RAD51sh-Surv clones revealed better survival than of LN229 cells whereas RAD51sh and RAD51sh-Surv (mock) cells were hypersensitive. RAD51sh-Surv (mock), as well as RAD51sh (not shown) underwent apoptosis, whereas RAD51sh-Surv clones were transiently arrested in G2 and entered G1. Since the survival of RAD51sh-Surv was lying over the survival of LN229 cells (for stained colonies, see Suppl. Fig. S3a), but repair itself was similar as of LN229, RAD51sh-Surv cells obviously must have progressed through the cell cycle with a certain amount of unrepaired DNA damage.

The ability of Surv-GFP to partially rescue the repair and enhance cell survival in the RAD51kd background was not due to switching to NHEJ, since inhibition of NHEJ using the DNA-PKcs inhibitor NU7026 did not influence viability differences upon TMZ (Suppl. Fig. S7a) and, also, did not affect the differences in colony formation between LN229-Surv and LN229-SurvNESmut cells (Suppl. Fig. S7b). In addition, upon TMZ the DNA-PKcs activity was rather reduced (Suppl. Fig. S7c).

To analyze whether TMZ differentially transcriptionally regulates important factors involved in resistance, qPCR analyses were performed (Suppl. Fig. S7d). The data revealed a significant repression of *Survivin* in SurvNES and RAD51sh cells and a missing transcriptional induction of *c-IAP2* in RAD51sh and RAD51sh-Surv cells. Since *c-IAP2* is a target of NF-κB, which plays an important role in the maintenance of TMZ-induced senescence [7], this might be a hint for missing senescence induction in these cells. This was further supported by marginal (RAD51sh) or absent (RAD51sh-Surv) senescence-associated secretory phenotype (SASP) activity, as shown by low or no induction of *IL6* and *IL8*, which was in complete contrast to LN-SurvNES cells showing huge *IL6*/*IL8* induction and a significant induction of *c-IAP2*. The missing senescence induction was further proven by ß-Gal staining (Suppl. Fig. S5d). Interestingly, whereas *RAD51* expression was reduced in LN229, LN-SurvNES and RAD51sh cells, TMZ did not alter *RAD51* expression in RAD51sh-Surv cells. A significant induction of *RAD51* in LN229-Surv cells was accompanied by protein stabilization, and a transient *p21* induction led to reduction in p21 protein expression (see Fig. 3d).

### Nuclear versus cytoplasmic Survivin reveals differential transcriptional processes upon TMZ

**Fig. 5:**
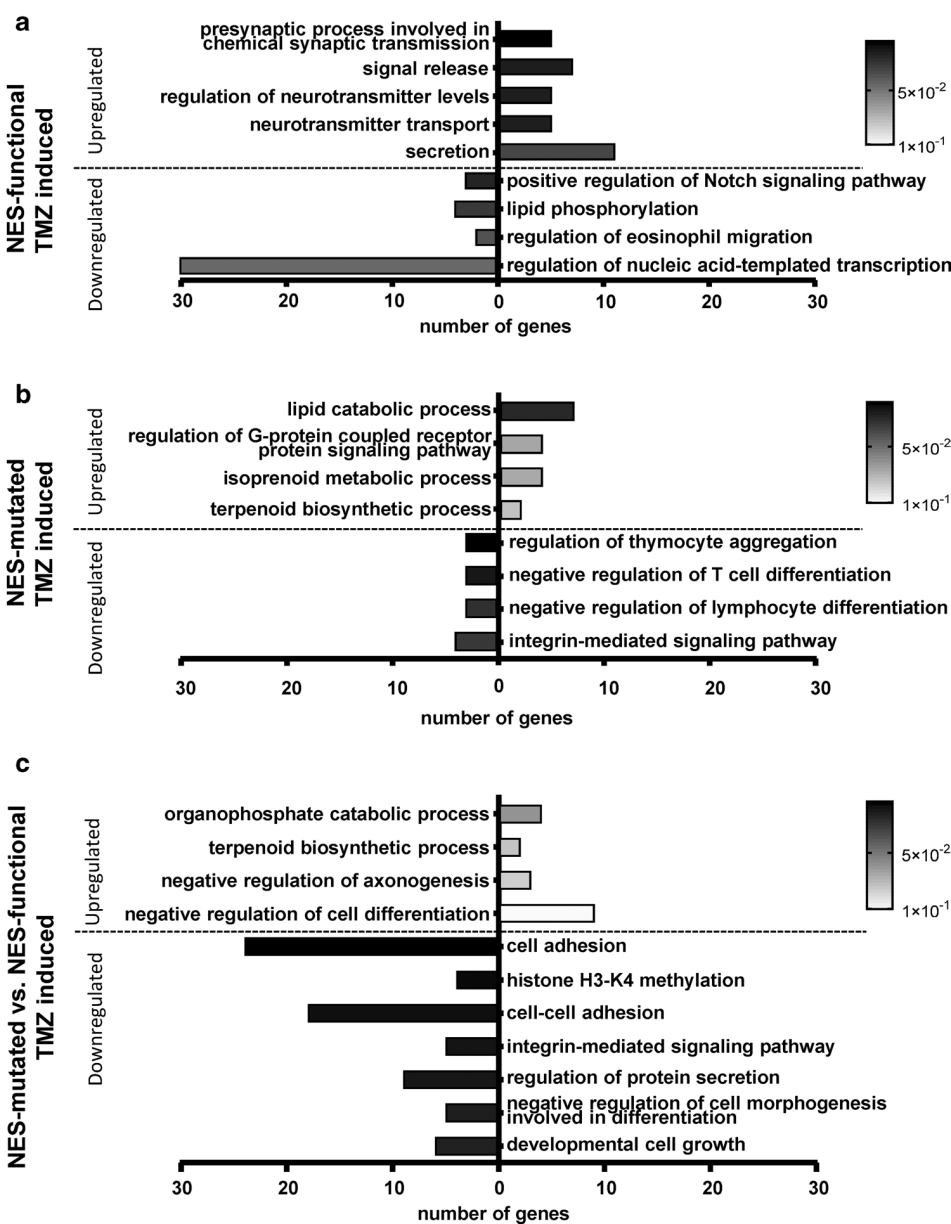
Biological processes (GO terms) associated with the different contrasts obtained from the transcriptomics dataset. Enrichment analyses were performed via DAVID, utilizing the BP_ALL subset of the GO database. *p* < 0.25 was used as the threshold for pre-filtering the input data, while an Ease of 0.1 was used for the results. **a** Comparison between TMZ-exposed (50 µM, 48 h) and unexposed clones with enhanced cytoplasmic Survivin expression and functional NES (Surv-GFP-D6) and **b** between TMZ-exposed and unexposed NES-mutated Survivin clones (SurvNESmut-GFP L2). **c** Normalized comparison of SurvNESmut-GFP-L2 (NES-mutated) to Surv-GFP-D6 (NES-functional) clones. Black-colored bars indicate high significance and white-colored less significance of depicted selected GO terms. The data represent two independent biological replicates in technical duplicates each of the two LN229 Survivin-expressing clones, Surv-GFP-D6 vs. SurvNESmut-L2

To examine whether TMZ induces genome-wide transcriptional changes in dependence of Survivin localization, transcriptomics analyses were conducted. The chosen p-value for pre-filtering was 0.25 and the data were subjected to GO term analysis. In glioma clones harboring cytoplasmic Survivin with functional NES, TMZ led to an increase in the processes involved in chemical synaptic transmission, neurotransmitter transport, regulation of neurotransmitter levels, signal release and secretion, all of which point towards alteration of neuronal signaling. The analysis of the downregulated genes showed e.g. associations with Notch signaling (Fig. 5a, for volcano plots, see Suppl. Fig. S8a), implicating regulation of glioma stemness [24] and reduction in secondary senescence [25]. In contrast, when the ectopically expressed Survivin was trapped in the nucleus, TMZ upregulated genes associated with processes involved in lipid catabolism, isoprenoid metabolism and terpenoid biosynthesis [26] indicating reduced tumor cell proliferation and growth [27]. Interestingly, the downregulated processes implicate effects on increased immune system and reduced integrin-mediated signaling [28] (Fig. 5b, Suppl. Fig. S8b).

Comparing the effects of TMZ on NES-mutated to NES-functional inter-clonally, in NES-mutated cells, the downregulated genes were associated with the biological processes (cell)-cell adhesion, related to matrix detachment and onset of apoptosis [29], histone H3K4 methylation and regulation of protein secretion, hinting at enhanced senescence and senescence-associated secretory phenotype (SASP), chromosome misalignments and segregation defects by putatively involved demethylases [30–33]. In contrast, the biological processes cell differentiation and negative regulation of axonogenesis were associated with the upregulated genes while developmental cell growth was found to be associated with the downregulated set, pointing against dedifferentiation, i.e., cancer cell stemness (Fig. 5c, Suppl. Fig. S8c). Collectively, based on a different subcellular localization of Survivin, TMZ triggers different gene expression patterns – however, putative biological significance of those transcriptionally regulated pathways must be investigated in the future.

### Impact of nuclear-trapped and cytoplasmic Survivin on TMZ response in glioblastoma xenografts

**Fig. 6:**
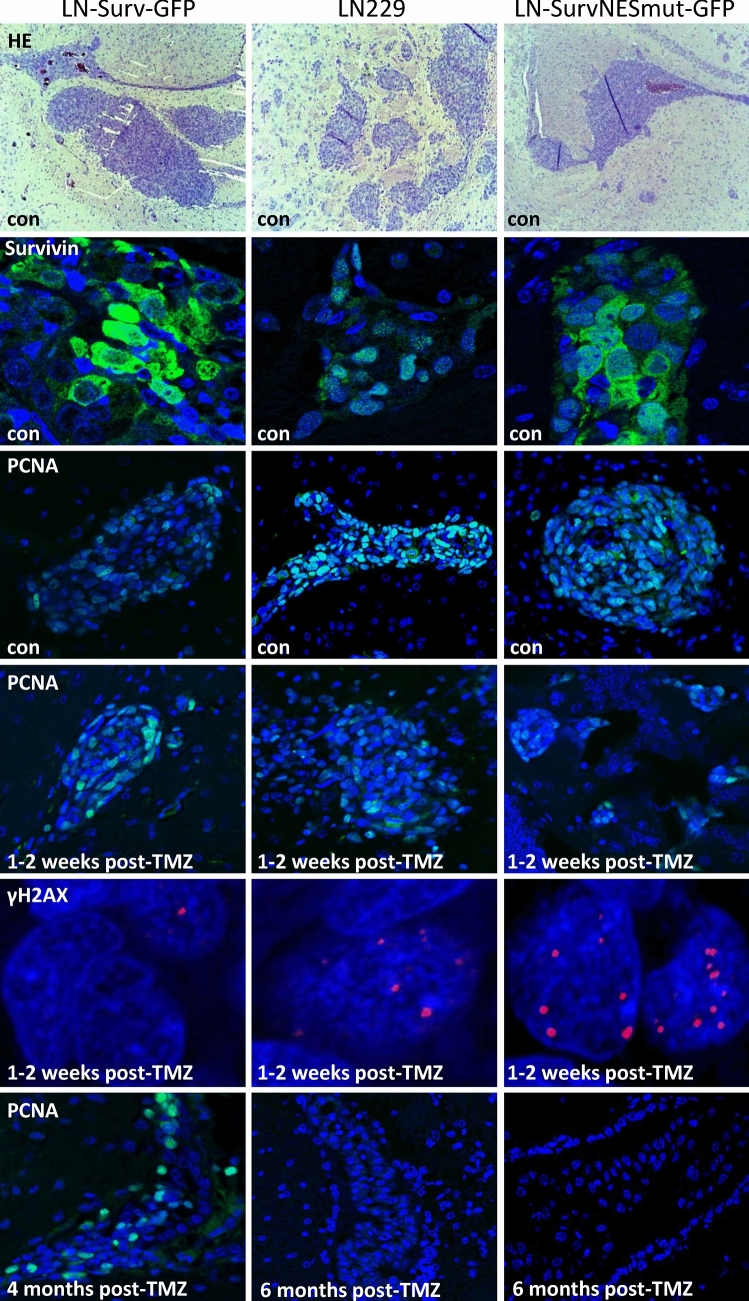
HE staining and immunofluorescence representative images of glioblastoma xenograft brain tissue sections in unexposed (control) and TMZ-exposed mice. TOPRO-3 (blue) was used to show nuclei. Antibodies used are listed in the Suppl. Table 2

**Fig. 7:**
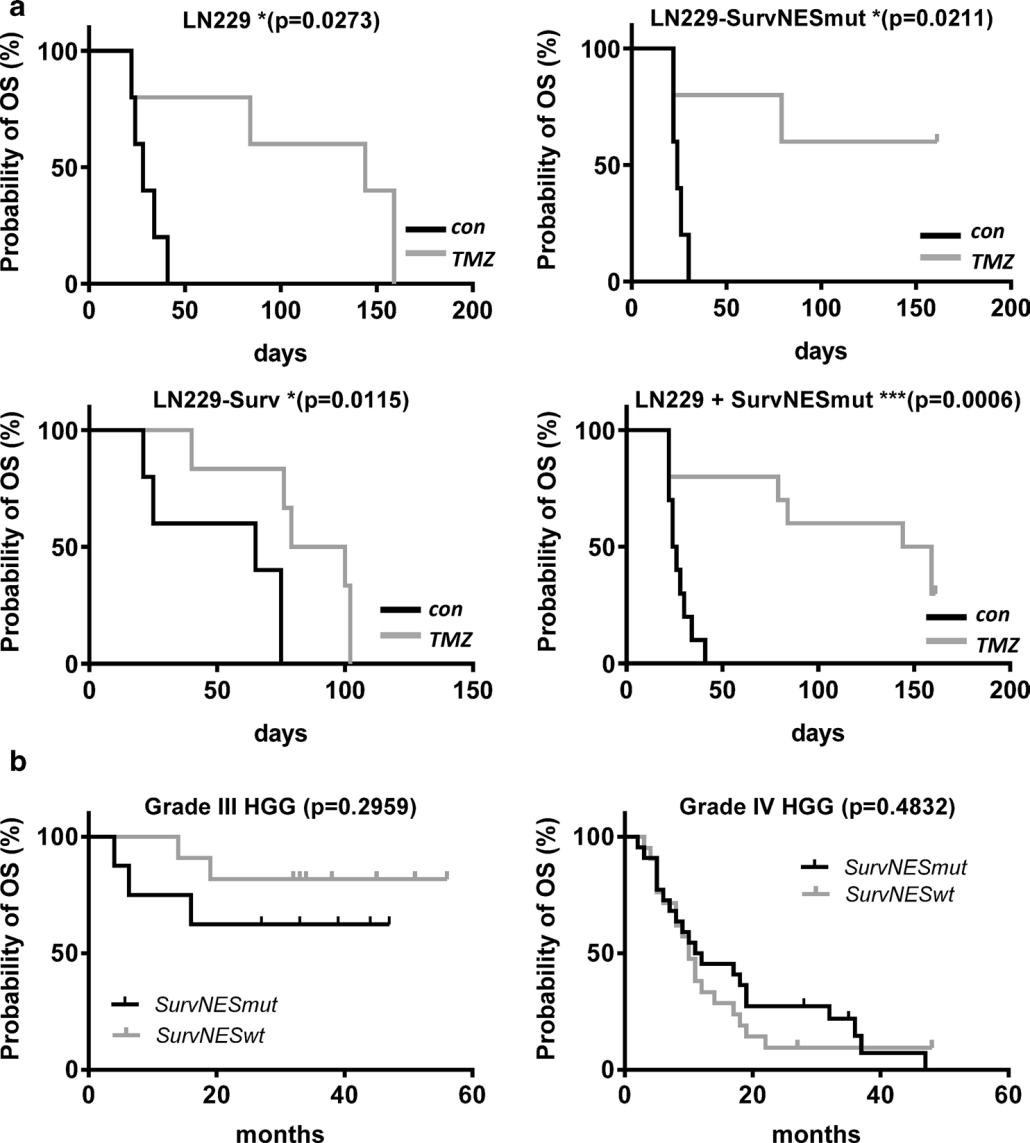
**a** Kaplan–Meier estimates of mice harboring Surv-GFP (clone D6), LN229 or Survivin-NESmut-GFP (clone L2) glioblastoma xenografts upon administration of TMZ. “LN229-SurvNESmut” (upper right graph) represents the estimation for the animal group with LN229-SurvNESmut implants and “LN229 + SurvNESmut” (lower right graph) represents the estimation for the two implant groups (LN229 and LN229-SurvNESmut) combined. The survival differences were tested for statistical significance by log-rank (Mantel-Cox) test (Graph Pad Prism 6.01) *Significant, ***highly significant. **b** survival curves of HGG patients in dependence of putative NES mutations. The survival differences in both groups were tested for statistical significance by log-rank (Mantel-Cox) test and were computed using SPSS 23

To verify our in vitro findings, we established an intracranial glioblastoma xenograft model. Xenografts with high nuclear localization of Survivin (SurvNESmut-GFP) exhibited similar growth rates compared to those with ‘normal’ nuclear localization (LN229) and in average grew faster to the ones with predominantly cytoplasmic Survivin (Surv-GFP), as shown by the weaker PCNA signal in the unexposed control, substantiating slower tumor cell proliferation (Fig. 6). One to two weeks after the end of TMZ exposure, the tumor growth (PCNA expression) of SurvNESmut-GFP xenografts was reduced. This can be explained by persisting DSBs (as shown by γH2AX foci) when compared to Surv-GFP xenografts, which still showed a strong PCNA signal and less DSBs (Fig. 6). Due to the efficient repair of DSBs in the Surv-GFP xenografts, the mice hardly responded to TMZ treatment and all mice had to be sacrificed preliminary. Dispersed PCNA staining was observed in the respective tumors. In contrast, TMZ-treated mice with SurvNESmut- or LN229-derived implants, which were killed at the scheduled end of the experiment at six months, were *bona fide* tumor free. Based on the in vitro results this indicates that also in vivo the abrogation of DSB repair in the tumor might enhance apoptosis and senescence. In line with this, Kaplan–Meier curves showed significantly better survival of TMZ-treated mice with tumors predominantly expressing nuclear Survivin (Fig. 7a). Hence, “LN229-SurvNESmut” (upper right graph) represents the estimation for the LN229-SurvNESmut implant group alone, whereas “LN229 + SurvNESmut” (lower right graph) represents the estimation for the implant groups LN229 and LN229-SurvNESmut combined. The p-value of the combined Kaplan–Meier estimates is highly significant (p = 0.0006) due to a higher number of animals. Thus, the data substantiate the in vitro findings, underpinning nuclear Survivin reinforces the positive response of gliomas to TMZ.

### Survivin distribution and screening for NES-inactivating somatic mutations in HGG samples

Apart from LN229, also other glioma cell lines (A172, U87) showed an enhanced nuclear expression of endogenous Survivin (Suppl. Fig. S9a). Further, a subset of 40 HGG patients’ samples was analyzed for a putative correlation between Survivin localization and survival. However, in all HGG patients’ tissues (grade III and IV) analyzed, Survivin was localized mainly in the nucleus (∼75–90% nuclear vs. 10–25% cytoplasmic), as shown by a representative example (Suppl. Fig. S9b). Thus, we performed pyrosequencing in all 86 HGG patient samples for known mutations in the NES to clarify whether NES mutations are occurring in HGG and whether a link with patients’ survival can be deduced. DNA, isolated from HGG samples, was thus sequenced for specific mutations in the NES of *BIRC5*. Mutations annotated in Suppl. Fig. S10a were described to cause a nuclear accumulation of Survivin in head and neck squamous cell carcinoma [13]. Pyrosequencing primers were set-up to identify two NES-inactivating transitions in genomic DNA (278 T > C and 292 C > T) and one silent transition in between (A288G). In none of the 86 tumors a 278 T > C transition was detected (Suppl. Fig. S10b). However, ∼50% of the patients showed a low frequency of 292 C > T transitions (< 13%) and in few cases a silent (A > G) transition (Suppl. Fig. S10c), indicating that only some cells within the tumor carry this C > T *BIRC5* NES mutation. These patients were analyzed for peculiarities in the survival. Patients with a detected C > T mutation frequency > 5% were designated as “noticeable”. Nevertheless, no significant difference in the OS was detected in grade III or grade IV glioma patients upon stratification for the *BIRC5*-NES mutation status (Fig. 7b). Thus, the sequencing data and IF/IHC staining let us deduce that despite the absence of 278 T > C mutations and a low frequency of 292 C > T mutations in the NES within our patients’ collective data, Survivin is nevertheless predominantly found in the nucleus of glioma cells.

## Discussion

Survivin’s biological function depends largely on its intracellular localization, and our data broaden those functions by a marked role of Survivin’s localization in senescence and DNA repair upon DNA damage induced by the anticancer drug TMZ. Thus, cytoplasmatic Survivin enhances reproductive survival of glioblastoma cells, whereas nuclear-trapped Survivin abrogates it. This reduction in clonogenic survival is due to onset of senescence. At early time points TMZ-triggered DDR is similar among the Survivin clones and the parental cells studied, irrespective of Survivin’s subcellular localization, except for γH2AX expression/foci, which are not only a part of DDR but also a marker for DSBs. However, at later times, TMZ-triggered p21 protein induction in SurvNESmut cells was sustained, as compared to LN229 and LN229-Surv cells, corresponding to prolonged *p21* transcriptional induction (Suppl. Fig. S7d). This led, on one hand, via CDK1 inhibition [7], to a stronger G2/M-arrest in SurvNESmut cells, as shown by the G2-marker CENP-F. On the other hand, it is known that p21 induction causes premature activation of the anaphase-promoting complex/cyclosome (APC/C), which is known to degrade cyclin B and other mitotic factors resulting in the transition from the G2-phase to a tetraploid (4n) G1-state without entering mitosis – so-called mitotic skipping [34, 35]. This was particularly pronounced in SurvNESmut cells, exhibiting more polyploid cells. Upon genotoxic stress *BIRC5* was shown to be transcriptionally repressed by p21 or E2F4 (DREAM) [36]. Although being repressed 48 h after TMZ exposure, *BIRC5* expression in LN229 and LN229-Surv cells recovered after additional 48 h, which was not the case for SurvNESmut cells (Suppl. Fig. S7d). Of note, the primer sequences used for the detection of *Survivin* locate to the 3’ UTR of the mRNA sequence and, therefore, only amplify the endogenous and not the exogenous *Survivin*. The reduction in endogenous *Survivin* expression correlates with the prolonged *p21* induction in SurvNESmut cells 96 h upon TMZ. Thus, TMZ triggers not only nuclear translocation of Survivin but also leads to reduced synthesis of the Survivin protein due to the transcriptional *BIRC5* repression. It was reported that glioma cells can escape from TMZ-induced senescence through modulation of CDK1/Survivin signaling [37]. Here, we could show that transcriptional *BIRC5* expression can recover in LN229-Surv cells upon TMZ, a step potentially leading to escape from senescence, and that these cells exhibit induced *RAD51* and *EXO1* transcriptional activity (Suppl. Fig. S7d), which acts against TMZ-induced senescence [7]. This is not the case in TMZ-exposed SurvNESmut cells, where *RAD51* and *EXO1* are repressed (Suppl. Fig. S7d) and senescence strongly induced.

Transcriptional repression of *RAD51* and *EXO1*, the latter involved in end-resection events during HR, also hints to a reduced/impaired repair capacity of cells with nuclear-trapped Survivin. This is substantiated by reduction in RAD51 protein expression, increased number of γH2AX foci and by strongly abolished HR activity in SurvNESmut cells. Significantly reduced HR activity was also reported when Survivin was silenced in breast cancer cells [38], or upon the indirect Survivin inhibitor YM155 and ionizing radiation in glioblastoma cells [39]. Due to strongly impaired HR activity in the NES-mutated cells, the persistent DSBs lead to formation of chromosome aberrations and most importantly, they trigger senescence and SASP, as shown by induction of *IL6* and *IL8*. This is highly important since, as a putative tumor-suppressing mechanism, SASP can reinforce the growth arrest by increasing ROS production and enhancing DDR [40]. In addition, SASP induces an inflammatory response and activates immune cells which eliminate senescent tumor cells [41, 42]. Accordingly, we observed a significantly higher NF-κB-dependent transcriptional induction of *c-IAP2* in the SurvNESmut than in LN229-Surv cells, which clearly unravel the potential of c-IAP antagonists to be putative senolytics and be administered to avoid recurrences [43]. Transcriptional differences between the clones, observed by RNA-Seq analyses and qPCR, fit in with the findings for the biological end-points senescence and chromosome instability. Also, very intriguing, cytoplasmic, not nuclear-trapped Survivin could significantly enhance HR activity and rescue TMZ-induced cytotoxicity upon RAD51 knockdown, again indicating Survivin’s putative pro-repair and anti-senescence role, as shown by highly reduced transcriptional activity of *IL-6/IL-8* in those clones. Importantly, this is not due to a switch to NHEJ, since no changes or even a reduction in DNA-PKcs activity upon TMZ was observed in all our clone variants (Suppl. Fig. S7c).

It was recently reported that upon ionizing radiation Survivin interacts with DNA-PKcs supporting NHEJ [23]. To analyze whether Survivin interacts with components of HR, we co-immunoprecipitated Survivin in the nuclear fraction of LN229 cells exposed to 50 µM TMZ for 48 h and conducted mass spectrometry-based proteome analysis. However, under those conditions, among the Survivin-interacting proteins neither of them belonged to DNA repair (HR or NHEJ) factors (Suppl. Fig. S11a-c). Also, upon TMZ, Survivin did not form repair foci with γH2AX or 53BP1/BRCA1/2 (not shown), what argues against its direct role in the repair of TMZ-induced DSBs. According to the volcano plots (Suppl. Fig. S11b) and the STRING analysis (Suppl. Fig. S11d), 53BP1 may biologically be enriched with the bait in a subpopulation of cells or it may present a low percentage among BIRC5 (Survivin) interactors, but statistically it's falling behind the thresholds we set. Thus, in case of TMZ a direct Survivin role in the repair is quite unlikely, since also NHEJ (DNA-PKcs activity) could be excluded. This is in accordance with the finding that O^6^-MeG induced DSBs are not repaired by NHEJ [8].

Opposite to a direct interaction with DNA repair an indirect effect on cell cycle and thereby senescence and clonogenic survival is more likely. We favor the hypothesis that reduced HR and thus enhanced DNA damage and senescence upon TMZ may be caused by altered CPC targeting, which certainly should be questioned and investigated in the future. Since Survivin is important for targeting the CPC to the centromere [44] and the Survivin–CRM1 interaction is essential for this process [10], enhanced expression of NES-mutated Survivin that could not bind its cognate CRM1 receptor could interfere with correct localization of the CPC. Of note, also CDCA5 (Cell Division Cycle Associated 5, Sororin), a factor important for cohesion maintenance on chromosomes [45] was found to be a putative (although weak) interactor of Survivin after TMZ treatment (Suppl. Fig. S11c, d). Incorrect targeting of the CPC and cytokinesis failure was already observed upon Survivin silencing [38]. This led to accumulation of cycling cells in the G2/M phase and increased exit from mitosis without cell division. Therefore, we suggest that analogous to the Survivin knockdown also NES-mutated Survivin leads to incorrect targeting of the CPC, resulting in cytokinesis failure, accumulation of polyploid cells and permanent G2-arrest (early genotoxin-induced senescence). This further reinforces transcriptional repression of *RAD51* and thereby leads to highly reduced HR activity, which results in persistent DSBs and the generation of chromosome aberrations, senescence progression and its maintenance. Of note, also silencing of INCENP induced polyploidization, apoptosis, and senescence in neuroblastoma cells, highlighting the importance of proper CPC localization for the prevention of senescence [46]. Opposite, overexpression of wt-Survivin might enhance proper targeting of the CPC complex and reduce cytokinesis failure and senescence, thereby lowering TMZ-induced RAD51 and HR repression, which we could observe at RNA and, particularly, at protein level, where under overexpression of Survivin, RAD51 was stabilized. Furthermore, this hypothesis is highly plausible because, in comparison to LN229 and LN-SurvNESmut cells, LN229-Surv cells exhibited a significantly higher number of mitotic cells after TMZ treatment, which were not aberrant as was often the case in SurvNESmut cells (see Suppl. Fig. S4d), and they showed significantly lower spontaneous and TMZ-induced level of stable chromosome aberrations (reciprocal translocations/inversions) (see Suppl. Table S4). In support, we observed a significant *RAD51* transcriptional activity as well as reduced *p21* transcription in cells with cytoplasmic Survivin while in the NESmut clone opposite was the case. Also, upon Survivin silencing, increased DSBs correlated with reduced HR and repression of *RAD51* and *EXO1* [38].

In various tumor types, nuclear Survivin was identified to be a favorable predictor [47], since its interaction with anti-apoptotic partners in the cytoplasm or in the mitochondria is reduced [48]. It is therefore assumed that nuclear-cytoplasmic-mitochondrial transport is crucial for the tumor-protective function of Survivin. Nevertheless, there are some conflicting data reporting nuclear Survivin to be associated with poor survival [49]. The discrepancies may be largely dependent on the tumor type or the examined biopsy (post-operative vs. post-treated), or the type of therapy (chemotherapy vs. radiotherapy), or be due to the different classification criteria defining nuclear and cytoplasmic Survivin. In glioblastomas, nuclear Survivin was shown to predict poor survival after radiotherapy [50] and combined radio-chemotherapy [51]—predictive value for TMZ therapy alone, in MGMT-negative tumors, has not been accessed yet. Based on the xenograft data from this study we want to emphasize that enriched nuclear Survivin localization is with high probability predictive of the positive outcome of TMZ therapy alone. In support, six patients from our collective, who despite MGMT-deficiency exhibited extremely short OS (< 4 months upon diagnosis), tended to bear tumors with a lesser amount (∼60%) of nuclear Survivin (Kaplan–Meier estimates not shown because of few patients). Since however those glioma patients also experienced standard radiotherapy which triggers NHEJ, we should keep in mind that TMZ effects have been (partially) masked by radiotherapy.

Beside glioblastoma cell lines, we also showed that endogenous Survivin is predominantly localized to the nucleus of HGG patients’ tissue, irrespective of putative NES mutations, which are very rare and did not correlate with patients’ survival. Survivin nuclear accumulation offers growth advantage over the cytoplasmic one, since xenografted tumors with nuclear Survivin grew faster. This becomes a double-edge sword in course of TMZ therapy since tumors with Survivin captured in the nucleus exhibit impaired HR-mediated DSB repair. Persistent DSBs give rise to severe clastogenic effects, leading to cell death and to maintenance of cellular senescence, characterized by SASP. Thus, Survivin compartmentalization appears to be an important predictive biomarker for alkylation drug-based glioblastoma therapy and manipulating Survivin subcellular localization towards permanent trapping in the nucleus would facilitate TMZ response of malignant gliomas. Specific anchoring of Survivin in the nucleus of glioma cells, e.g., using specific molecules binding the Survivin’s NES to prevent interaction with its related CRM1 receptor, or administration of small-molecule inhibitors, specifically interfering with cytoplasmic Survivin, would ameliorate TMZ response.

Overall, we provide the first evidence that cytoplasmic Survivin renders glioblastoma cells in vitro and in a xenograft model less vulnerable to TMZ by indirectly supporting DSB repair by HR and decreasing the chromosome aberration frequency and senescence, whereas nuclear-trapped Survivin induces the opposite response.

## Supplementary Information

Below is the link to the electronic supplementary material.

Supplementary file1 (PDF 5624 KB)

## Data Availability

The datasets generated during and/or analyzed during the current study are available from the corresponding author on reasonable request. Supporting data can also be found in the Supplement to this manuscript. Raw sequencing data have been  deposited in GEO (GSE154337).
