# Supplementary material for: Localization matters: nuclear-trapped Survivin sensitizes glioblastoma cells to temozolomide by elevating cellular senescence and impairing homologous recombination
Source: Cell Mol Life Sci. 2021 Jun 8;78(14):5587–604. doi: 10.1007/s00018-021-03864-0 (PMC8257519; doi:10.1007/s00018-021-03864-0)
Supplement: Supplementary file 1 — Supplementary file1 (PDF 5624 KB) [file 18_2021_3864_MOESM1_ESM.pdf]

Suppl. Table S1: List of primers used for real-time qPCR

qPCR Primer	Sequence (5‘-3‘)
53BP1-fw	AAACCCAACTGTGATGAG
53BP1-rev	AAGAGAAGGAAGAAGATACTT
BIRC3/c-IAP2-fw	TCACTCCCAGACTCTTTCCA
BIRC3/c-IAP2-rev	CCCCGTGTTCTACAAGTGTC
BIRC5/Survivin-fw	ATGACTTGTGTGTGATGA
BIRC5/Survivin-rev	GTTTGTGCTATTCTGTGAA
EXO1-fw	GCTGATAGAGGAAGGTATT
EXO1-rev	ATTGGACATTATCTCAAGATG
MGMT-fw	CTCTTCACCATCCCGTTT
MGMT-rev	AATCACTTCTCCGAATTTCAC
IL-6-fw	GCTGCAGGACATGACAACTC
IL-6-rev	AACAACAATCTGAGGTGCCC
IL-8-fw	CCAAACCTTTCCACCCCAAA
IL-8-rev	CTCTGCACCCAGTTTTCCTT
p21-fw	TACATCTTCTGCCTTAGT
p21-rev	TCTTAGGAACCTCTCATT
RAD51-fw	GGGGTGATCAGTTTCTGTTGC
RAD51-rev	GCACAATCATCTGCAAGTGGG
XIAP-fw	CCGAAGAGAAACCACATTT
XIAP-rev	CTGAGCCAGATCAAAGTATG
GAPDH-fw	CATGAGAAGTATGACAACAG
GAPDH-rev	ATGAGTCCTTCCACGATA
ACTB-fw	TGGCATCCACGAAACTACC
ACTB-rev	GTGTTGGCGTACAGGTCTT

Suppl. Table S2: List of antibodies used for western blot and immunofluorescence

Protein	Antibody	RRID	Company
53BP1	MAB3802	AB_2206767	Merck Millipore
Chk1	#2360	AB_10890862	Cell Signaling Technology
p-Chk1	#2341	AB_10693298	Cell Signaling Technology
GAPDH	sc-32233	AB_627679	Santa Cruz Biotechnology
γH2AX	ab81288	AB_1640564	Abcam
HSP90	sc-13119	AB_675659	Santa Cruz Biotechnology
IκBα	sc-371	AB_2235952	Santa Cruz Biotechnology
p21 (187)	sc-817	AB_628072	Santa Cruz Biotechnology
p-p53 (Ser15)	#9284	AB_628087	Cell Signaling Technology
PARP1	#551024	AB_394008	BD Biosciences
PCNA (PC10)	sc-56	AB_628110	Santa Cruz Biotechnology
RAD51	ab63801	AB_1142428	Abcam
Survivin	#AF886<br>71G4B7	AB_355684<br>AB_2063948	R&D Systems<br>Cell Signaling Technology
α-Tubulin	ab7291	AB_2241126	Abcam
HRP conjugated anti-mouse	KCB002	AB_10703407	Rockland Immunochemicals
HRP conjugated anti-rabbit	KCB003	AB_10702763	Rockland Immunochemicals
IRDye 680LT anti-mouse	926-68022	AB_10715072	LI-COR
IRDye 800CW anti-rabbit	925-32213	AB_2715510	LI-COR
Alexa Fluor 488 anti-mouse	#A11017	AB_2534084	Life Technologies
Alexa Fluor 488 anti-rabbit	#A11070	AB_2534114	Life Technologies
Cy^{TM}3-AffiniPure F(ab')$_2$ fragment goat anti-mouse IgG, fragment specific	115-166-006	AB_2338700	Jackson ImmunoResearch Laboratories

Suppl. Table S3: Experimental procedure according to NIH ARRIVE guidelines

<table>
<tr>
<td>Study design</td>
<td>1.</td>
<td>
a. We compared 6 groups of animals:
1. LN229 wildtype implanted, treated only with the vehicle (controls).
2. LN229 wildtype implanted, treated with temozolomide (5 mg/kg, 5 days a week for 4 weeks).
3. Implanted with Survivin-GFP transfected cells, treated with vehicle.
4. Implanted with Survivin-GFP transfected cells, treated with temozolomide.
5. Implanted with NESSurv-GFP transfected cells, treated with vehicle.
6. Implanted with NESSurv-GFP transfected cells, treated with temozolomide.
b. The experimental units were cages of animals (3-4 animals per cage, each animal with a different mark to be followed in our Pyrat software).
</td>
</tr>
<tr>
<td>Sample size</td>
<td>2.</td>
<td>
a. Two experimental units were allocated to each group (2 cages with 3 to 4 animals).
b. In this series of experiments, it is necessary to be able to distinguish between the overall survival and sensitivity of the three groups with different implants to temozolomide. With a power = 0.8 and alpha at one-sided question = 0.05, a significant difference between the groups would be possible with an assumed therapeutic effect leading to a difference in tumor volumes of >50% with a standard deviation of 33% with a group size of n = 8. Due to technical limitations, such as implantation accuracy during surgery and histological evaluability due to the growth form of the tumor, it is recommended to use a group size of n = 10, however based on the 3R principle, we decided on n=8 to reduce the number of animals, but still be able to obtain statistically significant differences.
</td>
</tr>
<tr>
<td>Inclusion and exclusion criteria</td>
<td>3.</td>
<td>
a. We followed the state of the animals using score sheets, which included the following criteria:
<table>
<tr><td>1.body weight, relative to the weight of the untreated control group</td><td>Score</td></tr>
<tr><td>-Unaffected or increase</td><td>0</td></tr>
<tr><td>-Reduction > 10%</td><td>10</td></tr>
<tr><td>-Reduction > 20%</td><td>20</td></tr>
<tr><td>2. general condition</td><td></td></tr>
<tr><td>- Skin pink, eyes shiny, body orifices clean</td><td>0</td></tr>
<tr><td>- Skin livid, eyes cloudy</td><td>5</td></tr>
<tr><td>- Eyes sunken cloudy, clogged or moist body orifices</td><td>10</td></tr>
<tr><td>- animal feels cold, eyes closed, breathing noises, bluish mucous membranes</td><td>20</td></tr>
<tr><td>3. spontaneous behavior</td><td></td></tr>
<tr><td>- alert, curious, sitting up, nimble movements</td><td>0</td></tr>
<tr><td>- unusual behavior, restricted or reduced movement or overactivity, reduced exploration behavior</td><td>5</td></tr>
<tr><td>- Isolation, apathy, incoordination, marked stereotypies or hyperkinetics</td><td>10</td></tr>
<tr><td>- automutilation, necrosis, rectal prolapse</td><td>20</td></tr>
<tr><td>4. trial-specific criteria</td><td></td></tr>
<tr><td>-Ataxia, head tilt onset</td><td>5</td></tr>
<tr><td>-Ataxia, constant head tilt</td><td>10</td></tr>
<tr><td>Convulsions, paralysis, seizures, paresis, plegia, vocalization, tremor</td><td>20</td></tr>
</table>
b. Three animals were excluded, because the tumors grew in the ventricle, and the animals died before the therapy started. During the therapy, 4 mice were terminated either because of neurological symptoms or other health issues and were excluded from the analysis.
c. For the survival analyses the values for the experimental groups were:
LN229 controls n=7
LN229 treated with TMZ n=5
Survivin-GFP controls n=7
Survivin-GFP treated with TMZ n=8
NESSurv-GFP controls n=7
NESSurv-GFP controls n=7
</td>
</tr>
</table>

Randomization	4.	a. We used randomization based on body weight, since the provided animals varied in body weight at the time of the implantation from 21 to 27 g. We distributed in each unit (cage) animals with lower and higher body weight.<br>b. To avoid confounders the location of the cages in the thermostat during maintenance and the order of treatments and measurements were randomly changed.
Blinding	5.	Different stages of the experiment were conducted by different investigators:<br>• Implantations by Dr. Ella Kim<br>• Body weight measurement, health status determination, TMZ treatment, termination and organ removal by Dr. Teodora Nikolova<br>• Tissue slides preparation and immunohistochemistry by Birgit Rasenberger<br>• Data analysis and outcome assessment by Dr. Maja T. Tomicic
Outcome measures	6.	a. The most important endpoint, which we assessed was the survival of the experimental animals after tumor development without or with temozolomide treatment. Additionally, tumor growth of the tumors originating from the different implanted glioblastoma cell lines was assessed on immunohistochemical preparations stained for proliferation markers (PCNA)
Statistical methods	7.	Kaplan-Meyer estimates: log-rank test (Mantel-Cox test), Graph Pad Prism 6.01
Experimental animals	8.	a. Immunodeficient mice (strain NMRI-*Foxn1*$^{nu/}$nu), females, 6 weeks old at the time of arrival at the TARC facility, body weight 21-27 g. After acclimatization period of 2 weeks, they were implanted at the age of 8 weeks.<br>b. The animals were purchased from Charles River Europe and were not used in any previous procedures.
Experimental procedure	9.	Animal were maintained in thermostat and experiments were conducted at the Translational Animal Research Center (TARC) of the Johannes Gutenberg University Medical Center Mainz. Before implantation, mice were acclimatized for 2 weeks (see p.8), the implantations were performed. The animals were first anesthetized by i.p. injection of a ketamine/xylazine mixture in 1xPBS=phosphate salt buffer (ketamine 140 mg/xylazine 10 mg/kg body weight; application volume 0.01ml/g). The anesthetized mouse is positioned in the stereotactic frame in the thoracic position by hooking the mouse's incisors into the bite bar of the snout holder and tightening the nasal clamp over the snout and tightening the nasal clamp over the snout, ensuring that the mouse's head is exactly horizontal. the head of the mouse is exactly horizontal. The position of the animal is adjusted so that the tips of the ear supports are at the caudal end of the ear canal. Once the position of the skull is adjusted caudally, the bite splint is attached to the stereotaxic frame.<br>After adjusting the height of the ear bars, they are advanced into the caudal portion of the ear canal and secured so that the head of the mouse is exactly horizontal and immovable when touched by the is immovable when touched by the finger. After the ear rods are in place, the animal is monitored for signs of respiratory distress. The scalp was disinfected and after opening the scalp rind through a longitudinal 2-3 mm incision, the bregma was identified. The drill hole site was 3.0 mm lateral to the bregma and was first marked on the bone by a small score with a sterile cannula. A hole was then drilled through the calvaria at the marked site using a microdrill. A sterile hollow needle is then screwed into this hole exactly perpendicular to the skull calvaria of the mice. The following stereotactic coordinates are used in relation to the bregma 1 mm (anteroposterior axis), 3 mm (lateral axis), 2.5 mm (vertical axis). Through this needle, tumor cells in a total volume of 3 µl were injected into the basal ganglia of the animals over 3 minutes using a special micro-syringe (model Hamilton, type 702). The needle was then slowly removed over several stages and the skin is sealed with a skin adhesive. The ear supports were loosened, the mouse removed from the stereotactic apparatus and placed on a heating pad set to 37°C until the animal regains consciousness. The animals were inspected each day and three weeks post implantation, temozolomide treatment was started. Temozolomide was purchased from Sigma-Aldrich, diluted in DMSO at a concentration of 10 mg/ml, aliquoted and stored at -80°C. For treatment, temozolomide was quickly thawed and diluted in sterile 0.9 % NaCl to a final concentration of 1 mg/ml. Mice were injected i.p. with 5 mg/kg Body weight 5 days a week for 3 weeks.

<table>
<tr><td></td><td></td><td></td></tr>
<tr><td>Results</td><td>10.</td><td>Body weight of the mice during the experimental procedure:
<table>
<tr><td></td><td>LN229 wt</td><td>NESSurv-GFP</td><td>Survivin-GFP</td></tr>
<tr><td>implantation</td><td>23,1</td><td>23,5</td><td>24,0</td></tr>
<tr><td>start of TMZ</td><td>27,4</td><td>26,4</td><td>26,7</td></tr>
</table>
<table>
<tr><td></td><td>LN229</td><td>LN229</td><td>NESSurv</td><td>NESSurv</td><td>SurvGFP</td><td>SurvGFP</td></tr>
<tr><td>treatment</td><td>control</td><td>TMZ</td><td>control</td><td>TMZ</td><td>control</td><td>TMZ</td></tr>
<tr><td>1 week</td><td>27,7</td><td>26,0</td><td>25,9</td><td>26,8</td><td>27,2</td><td>25,1</td></tr>
<tr><td>2 weeks</td><td>29,0</td><td>25,6</td><td>25,4</td><td>26,4</td><td>27,6</td><td>26,9</td></tr>
<tr><td>3 weeks</td><td>29,3</td><td>26,3</td><td>24,6</td><td>27,0</td><td>27,9</td><td>27,2</td></tr>
</table>
For the final results, see Fig. 7</td></tr>
</table>

Suppl. Table S4: Types of chromosome aberrations in unexposed (control) and TMZ-exposed glioblastoma cell clones

Variant	No. Cells	*g'*	*g''*	*br'*	*br''*	*Tri*	*Q*	*D*	*DD*	*dic/ring*	recTr /inv	Total CA	Total CA-recTr	Σ Chrom.	No.Chr. per cell	CA per Chrom.	CA /cell	Cells with CA (%)	Cells with CA-recTr (%)	Cells with ind. CA (%)
LN229 con	50	1	0	2	4	0	0	0	0	0	12	18	6	3735	74,70	0,001606	0,12	34	22	
LN229 TMZ15	50	6	0	25	5	3	1	3	0	3	10	50	40	3758	75,16	0,010644	0,80	60	50	26
D6 con	50	4	1	17	0	0	0	0	0	0	0	17	17	3728	74,56	0,004560	0,34	26	26	
D6 TMZ15	50	7	0	40	2	5	0	0	0	3	0	50	50	3697	73,94	0,013524	1,00	56	56	30
NES con	50	0	3	0	0	0	0	0	0	1	8	9	1	3728	74,56	0,000268	0,02	22	14	
NES TMZ15	50	7	0	63	11	2	0	0	0	4	13	93	80	3759	75,18	0,021282	1,60	80	67	58

Variant	No. Cells	*g'*	*g''*	*br'*	*br''*	*Tri*	*Q*	*D*	*DD*	*dic/ring*	recTr /inv	Total CA	Total CA-recTr	Σ Chrom.	No.Chr. per cell	CA per Chrom.	CA /cell	Cells with CA (%)	Cells with CA-recTr (%)	Cells with ind. CA (%)
LN229 con	50	2	0	7	4	1	0	0	0	1	15	28	13	3790	75,80	0,003430	0,26	42	27	
LN229 TMZ15	50	3	0	24	11	11	7	2	0	1	34	90	56	3776	75,52	0,014831	1,12	84	50	42
D6 con	50	4	0	6	1	0	0	0	0	1	4	12	8	3760	75,20	0,002128	0,16	20	16	
D6 TMZ15	50	2	0	22	6	5	1	0	0	1	3	38	35	3713	74,26	0,009426	0,70	48	45	28
NES con	50	1	0	11	6	0	0	0	0	0	15	32	17	3740	74,80	0,004545	0,34	40	25	
NES TMZ15	50	3	0	49	2	6	3	1	0	2	29	92	63	3766	75,32	0,016729	1,26	88	59	48

Variant	No. Cells	*g'*	*g''*	*br'*	*br''*	*Tri*	*Q*	*D*	*DD*	*dic/ring*	recTr/ inv	Total CA	Total CA-recTr	Σ Chrom.	No.Chr. per cell	CA per Chrom.	CA /cell	Cells with CA (%)	Cells with CA-recTr (%)	Cells with ind. CA (%)
LN229 con	50	3	0	9	3	0	0	0	0	0	19	31	12	3780	75,60	0,003175	0,24	42	23	
LN229 TMZ15	50	11	0	58	4	4	4	0	0	4	10	84	74	3705	75,61	0,019973	1,51	84	74	51
D6 con	50	0	0	2	2	0	0	0	1	0	3	8	5	3786	75,72	0,001321	0,10	20	17	
D6 TMZ15	50	2	0	17	1	6	2	0	3	1	3	33	30	3795	75,90	0,007905	0,60	48	45	28
NES con	50	1	1	11	3	1	0	0	0	2	11	28	17	3783	75,66	0,004494	0,34	40	29	
NES TMZ15	50	6	0	105	12	9	19	4	0	1	16	166	150	3717	74,34	0,040355	3,00	88	72	43

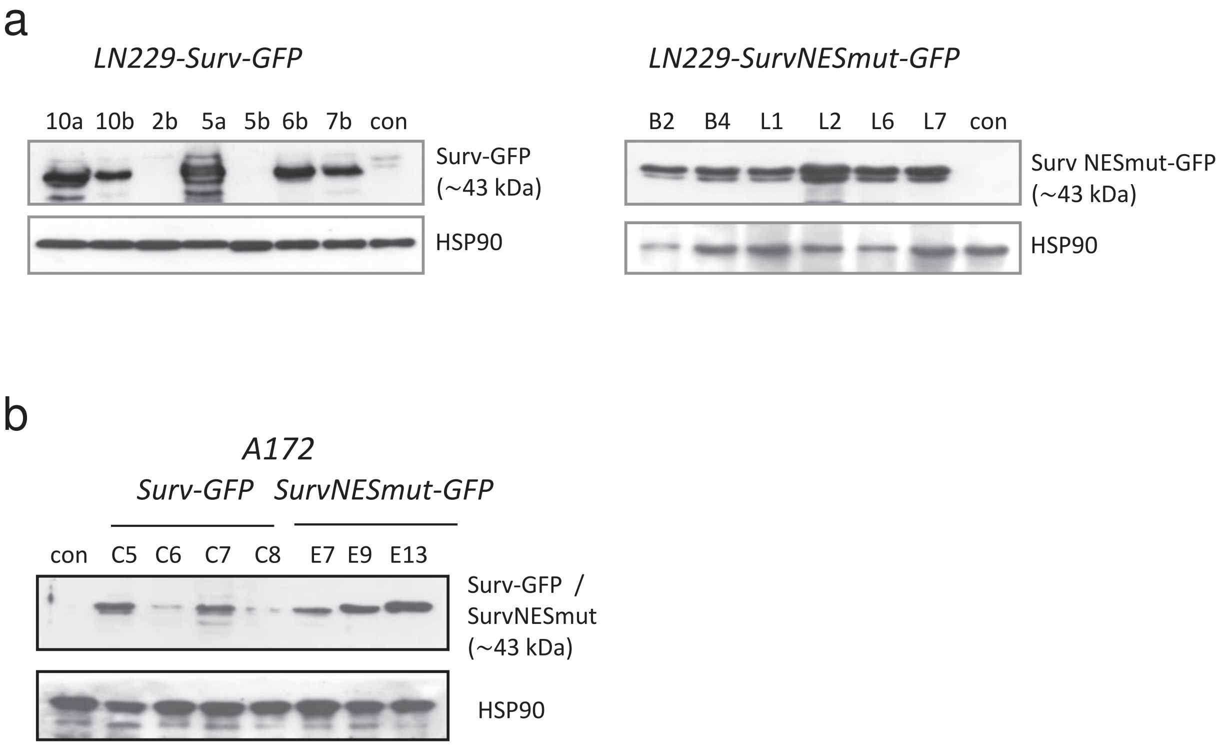

Suppl. Figure S1 a-b Western blot analysis of different stable LN229 and A172 cell clones, respectively, expressing the intact Survivin-GFP and mutated Survivin NESmut-GFP fusion protein. HSP90 was used as loading control

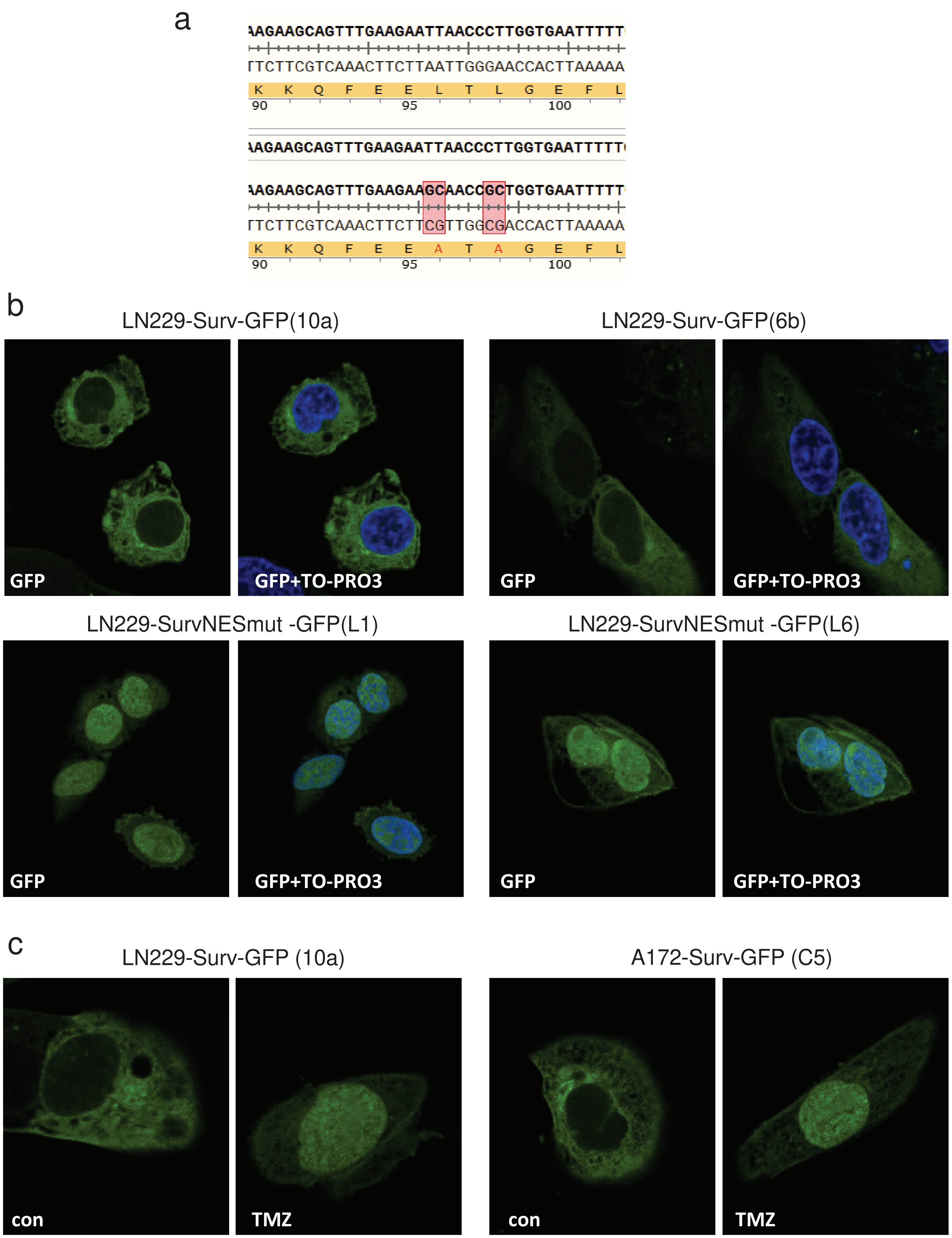

Suppl. Figure S2 a Alignment of BIRC5 CDS (NM_001168.2) and data obtained from sequencing of the pcDNA 3.1 SurvNESmut-GFP plasmid. Sequences were aligned with SnapGene (Trial Version). **b** Immunofluorescence staining showing distribution and localization of Survivin-GFP in different LN229 cell clones (clones 10a and 6b, upper panel) and of Survivin NESmut-GFP (clones L1 and L6, lower panel). The nuclei were stained with TO-PRO3 (depicted in blue). **c** Immunofluorescence staining showing nuclear translocation and accumulation of Survivin-GFP in LN229 (clone 10a) and A172 (clone C5) after exposure to 50 µM TMZ

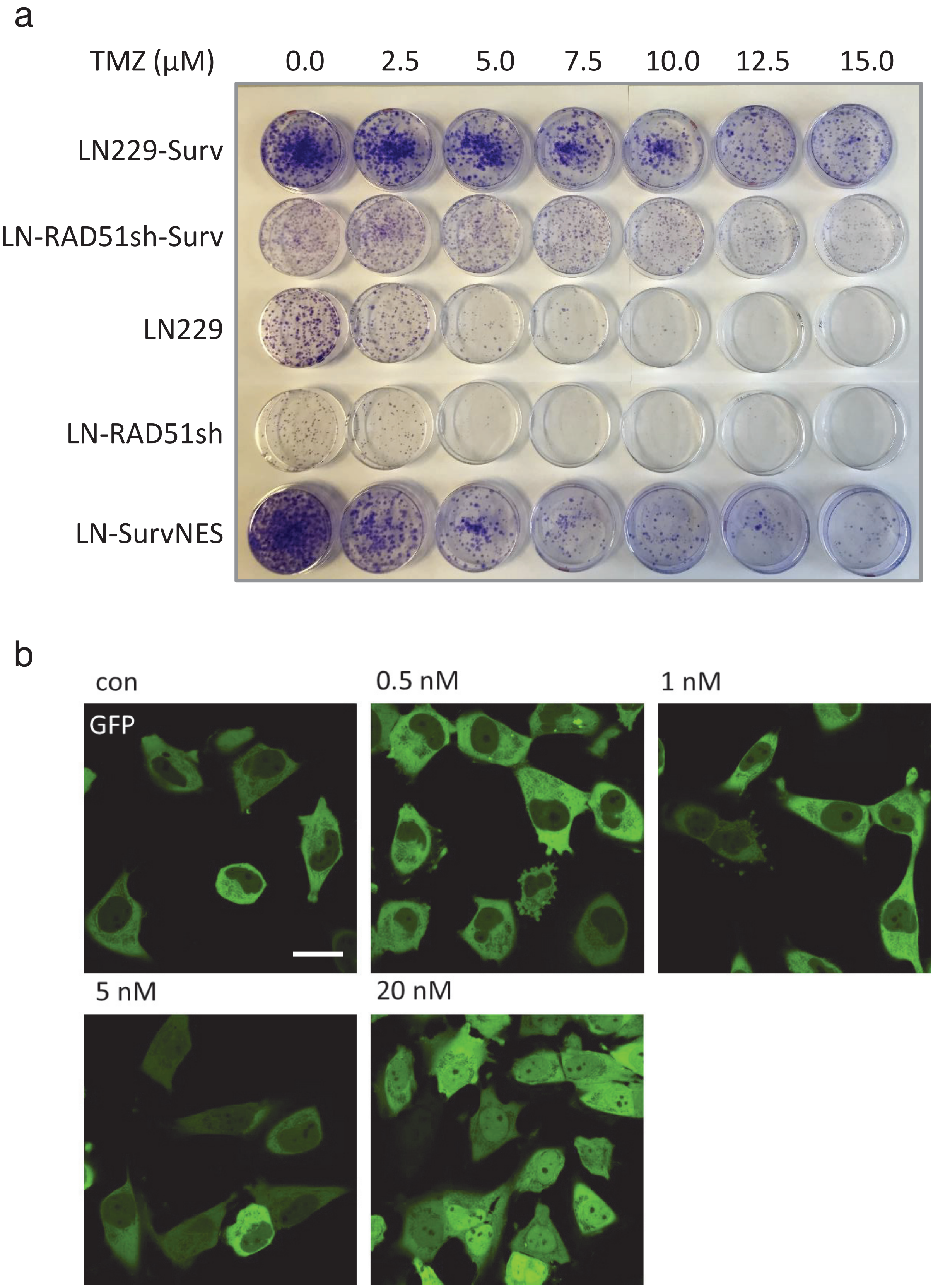

Suppl. Figure S3 a Crystal violet staining of colonies in 6-cm dishes for different TMZ-exposed LN229-derived clones. **b** Live cell imaging of LN229 Surv-GFP cells 60 min after treatment with different concentrations of the CRM1 inhibitor LMB. Cells were seeded in 4-well Tissue Culture Chambers (Sarstedt, Nümbrecht) with cover glass bottom for high-resolution microscopy. For imaging, supplemented medium without phenol red was used. Images were acquired on an LSM 710 (Carl Zeiss GmbH) using a Plan-Apochromat 63x/1.40 Oil DIC objective. GFP fluorescence is shown in green. Scale bar equate 20 µm

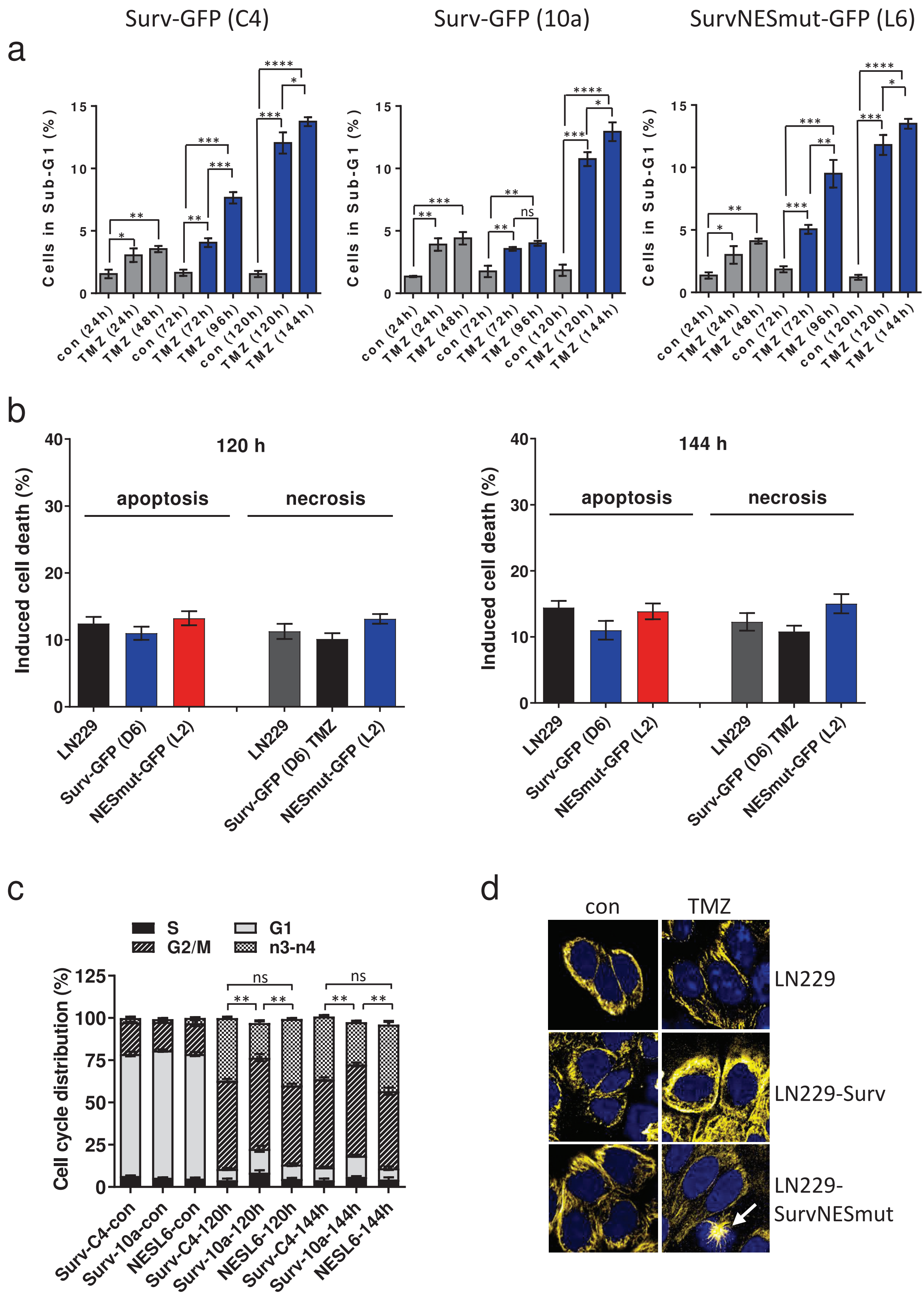

Suppl. Figure S4 Flow cytometric analysis. **a** Induction of cell death (SubG1) upon exposure of LN229 and the LN229 Survivin expressing clones (10a, C4 and L6) to 50 µM TMZ to 50 µM TMZ. Test for statistical significance was performed by Two-Way ANOVA with Bonferroni correction. $p^* \leq 0.05$ statistically significant, $p^{**} \leq 0.01$ very significant, $p^{***} \leq 0.005$ highly significant and $p^{****} \leq 0.001$ most significant; ns, not significant. **b** Induction of early and late apoptosis/necrosis in LN229 cells and different clones (D6, 10a, L2, L6) upon exposure to 50 µM TMZ for 120 and 144 h, measured by annexin V-APC/PI double staining; non-labeled = not significant. **c** Cell cycle distribution upon exposure of LN229 and the LN229 Survivin expressing clones (10a, C4 and L6) to 50 µM TMZ. Test for statistical significance was performed by Two-Way ANOVA with Bonferroni correction. $p^{**} \leq 0.01$ very significant; ns, not significant. **d** α-tubulin staining in LN229 and derived Survivin clones in unexposed controls and cells exposed to 50 µM TMZ for 48 h. The arrow shows an aberrant metaphase. α-tubulin, anti-goat Cy3 (shown in yellow)

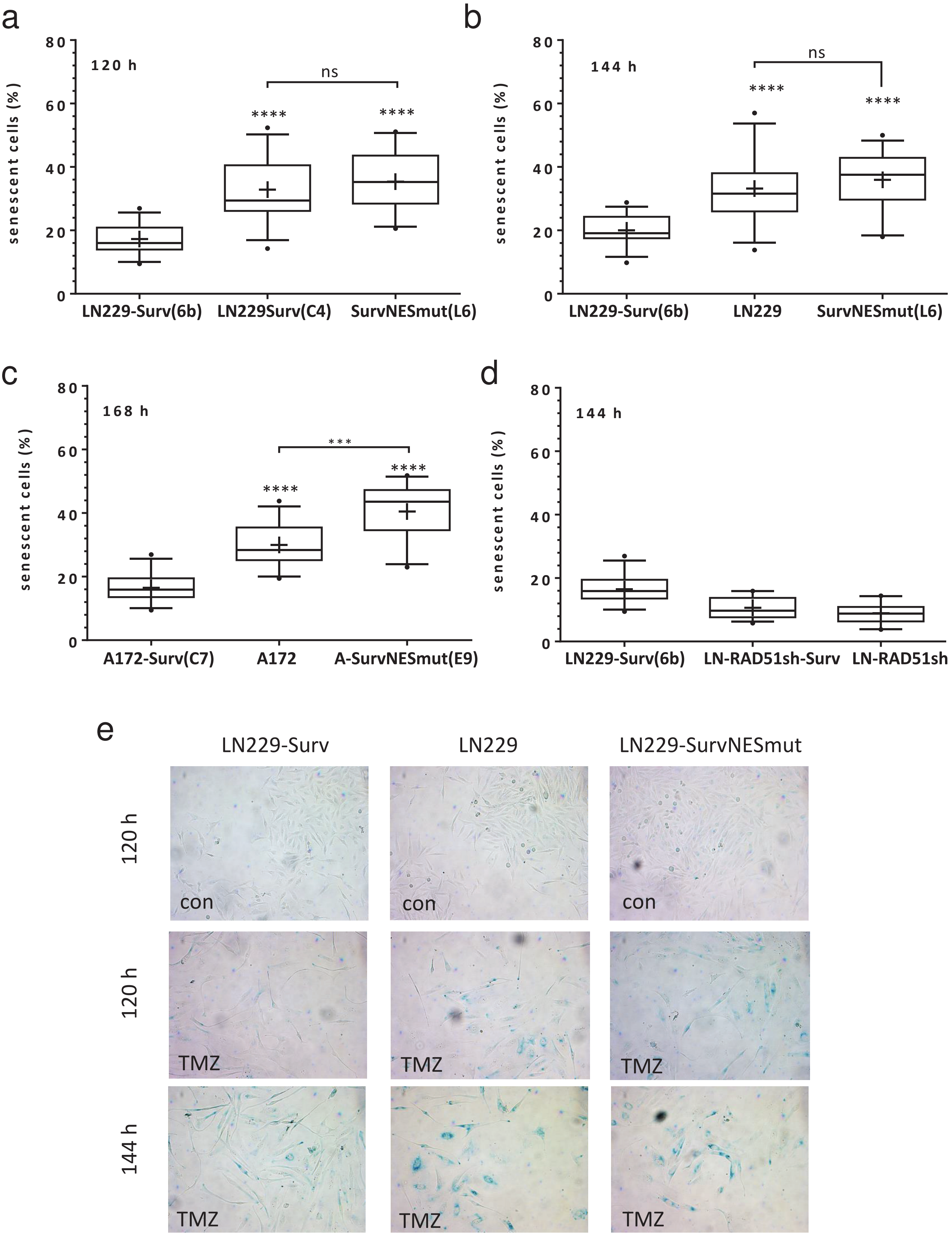

Suppl. Figure S5 a-d Box-plots showing senescence induction as fraction of SA-β-Gal positive cells after treatment with 50 μM TMZ. All time-points (120, 144 and 168 h) represent three independent experiments. Between 255 and 568 cells were counted for each group in each independent experiment (n=3). Whiskers indicate 5th and 95th percentile, with boxes representing first, second (median) and third quartile (from top to bottom). Geometric means are marked with "+". Outliers (values out of 5 – 95 percentile range) are marked as "•". Test for statistical significance was performed by One-Way ANOVA with Bonferroni post hoc analysis. $p^* \leq 0.05$ statistically significant, $p^{**} \leq 0.01$ very significant, $p^{***} \leq 0.005$ highly significant and $p^{****} \leq 0.001$ most significant; ns, not significant. **e** Representative bright field images of different LN229 cells stained for SA-β-Gal activity. Positive cells (strong blue) and total cells were counted on each image

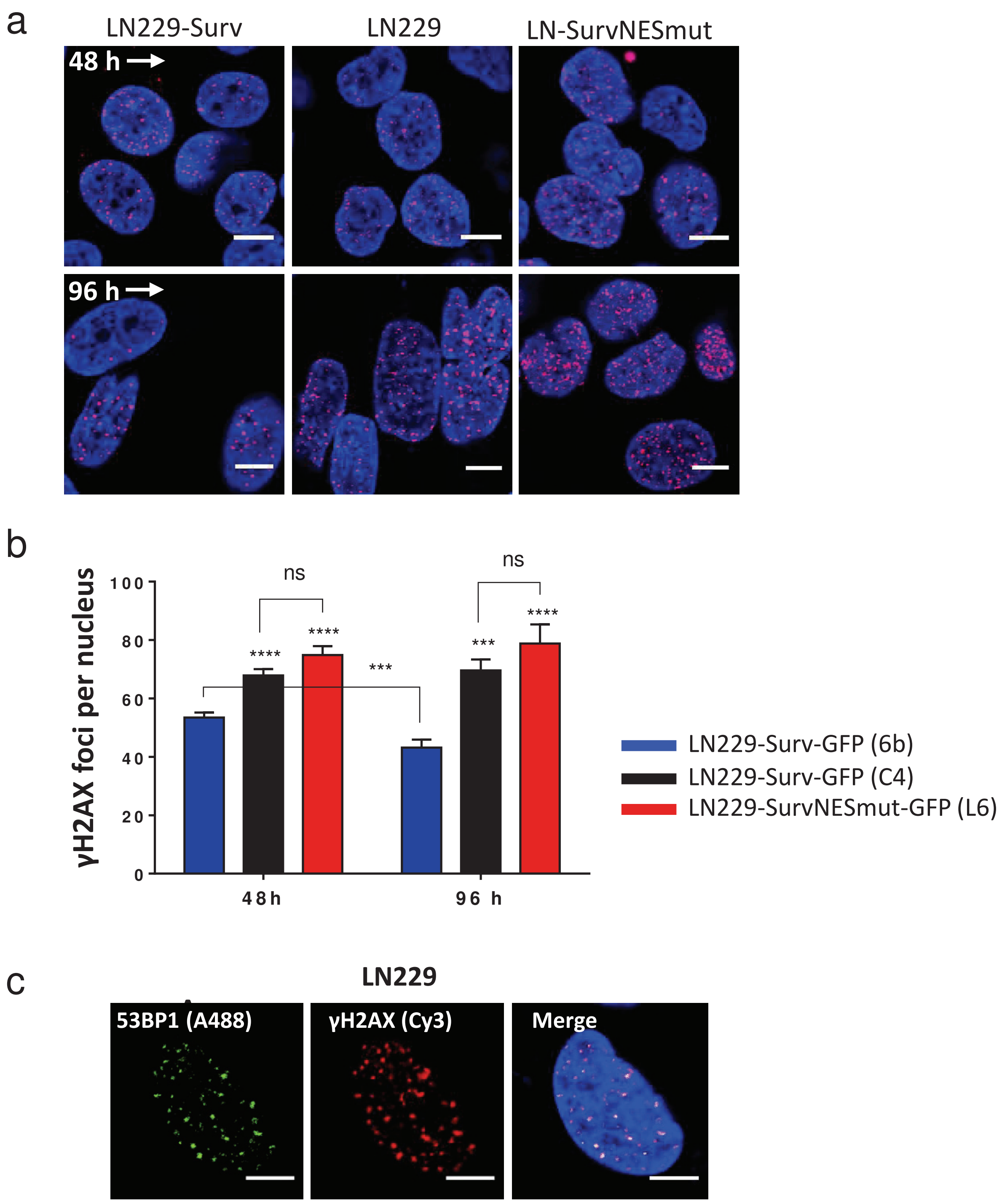

Suppl. Figure S6 a Immunofluorescence images of γH2AX foci (red) in LN229 cell clones upon treatment with 50 µM TMZ for different time points. TOPRO-3 (blue) was used to show nuclei. Scale bars equate 10 µm. **b** Summary of γH2AX foci formation per nucleus in Surv-GFP cell clones after treatment with 50 µM TMZ. At least 35 nuclei at each time point were evaluated. Error bars indicate SEM. Test for statistical significance was performed by One-Way ANOVA with post hoc analysis. p-values (*) indicated above each column were calculated between LN229 Surv-GFP (blue) and the corresponding column. Other comparisons are indicated above the bars. **c** Immunofluorescence images showing co-localization of 53BP1 and γH2AX, 96 h after TMZ treatment (50 µM). 53BP1 was stained with A488-conjugated (green) and γH2AX with Cy3-conjugated (red) secondary Ab. TO-PRO3 was used to show nuclei (blue). Scale bars equate 10 µm

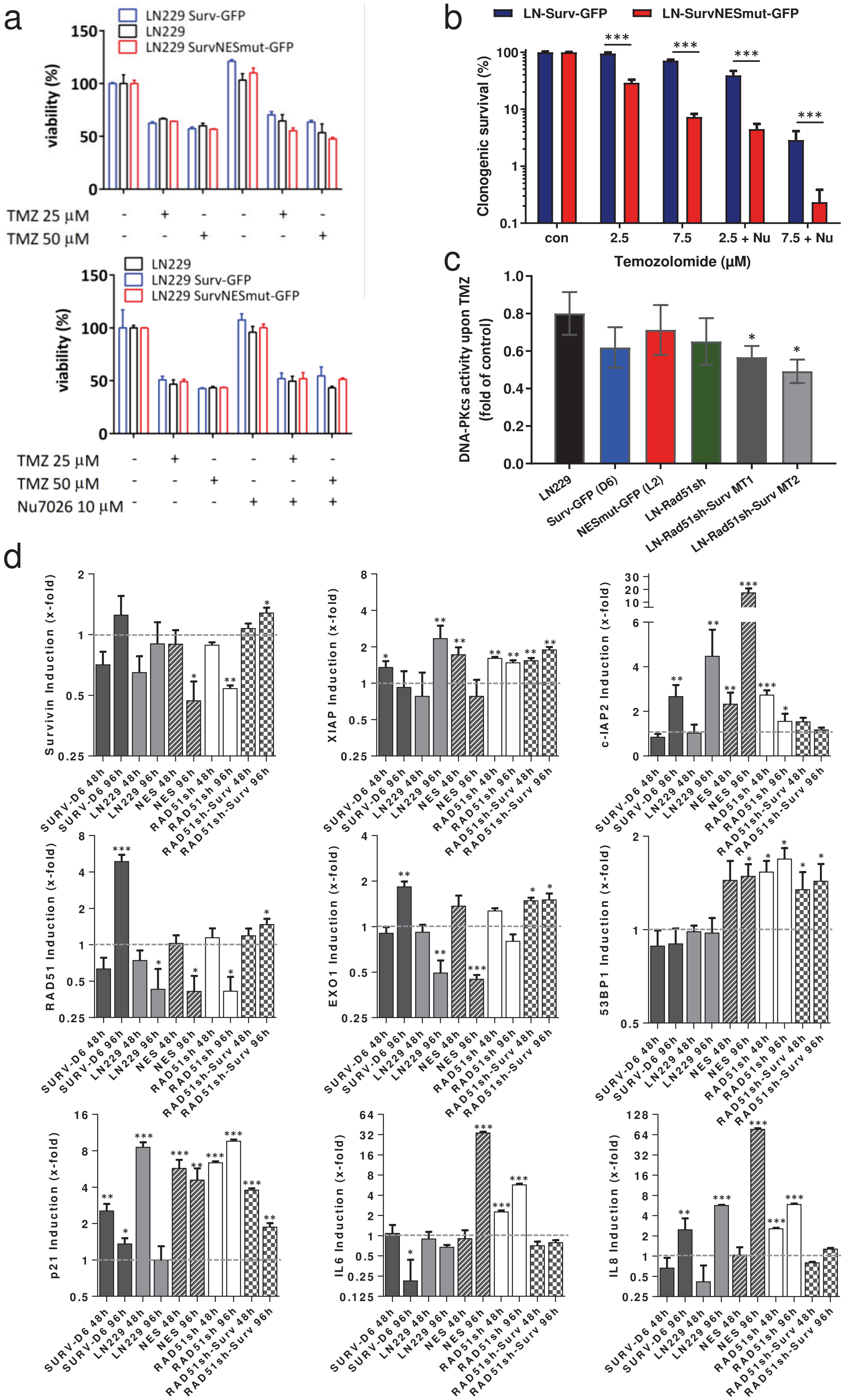

Suppl. Figure S7 a MTT cell viability assay of Survivin-expressing cell clones 72 h and 96 h after single and combination treatment with TMZ and the DNA-PK inhibitor Nu7026. Cellular viability was normalized to control treated cells (DMSO). Experiment was performed in technical triplicates for each time point. Error bars indicate the SD. **b** Colony formation assay of Survivin cell clones for different concentrations of TMZ and upon co-treatment with 10 µM Nu7026. Surviving cells were normalized to control (%) and plotted in a semi-logarithmic graph. Data of three independent experiments (N = 3) are shown with error bars indicating SD. **c** DNA-PK activity assay upon treatment of different LN229 cell variants to 50 µM TMZ for 72 h. Data of two independent experiments (N=2) in duplicates are shown with error bars indicating SD. **d** qPCR of transcriptional activity upon 50 µM TMZ after 48 and 96 h. Three independent experiments (N=3) in triplicates ± SD, comparing differences between treated cells and untreated control (set to 1, dotted line) are shown. **b, c, d** Data were statistically analyzed using Student's t test. $p^* \leq 0.1$ significant, $p^{**} \leq 0.01$ very significant, $p^{***} \leq 0.001$ highly significant; non-labeled = not significant

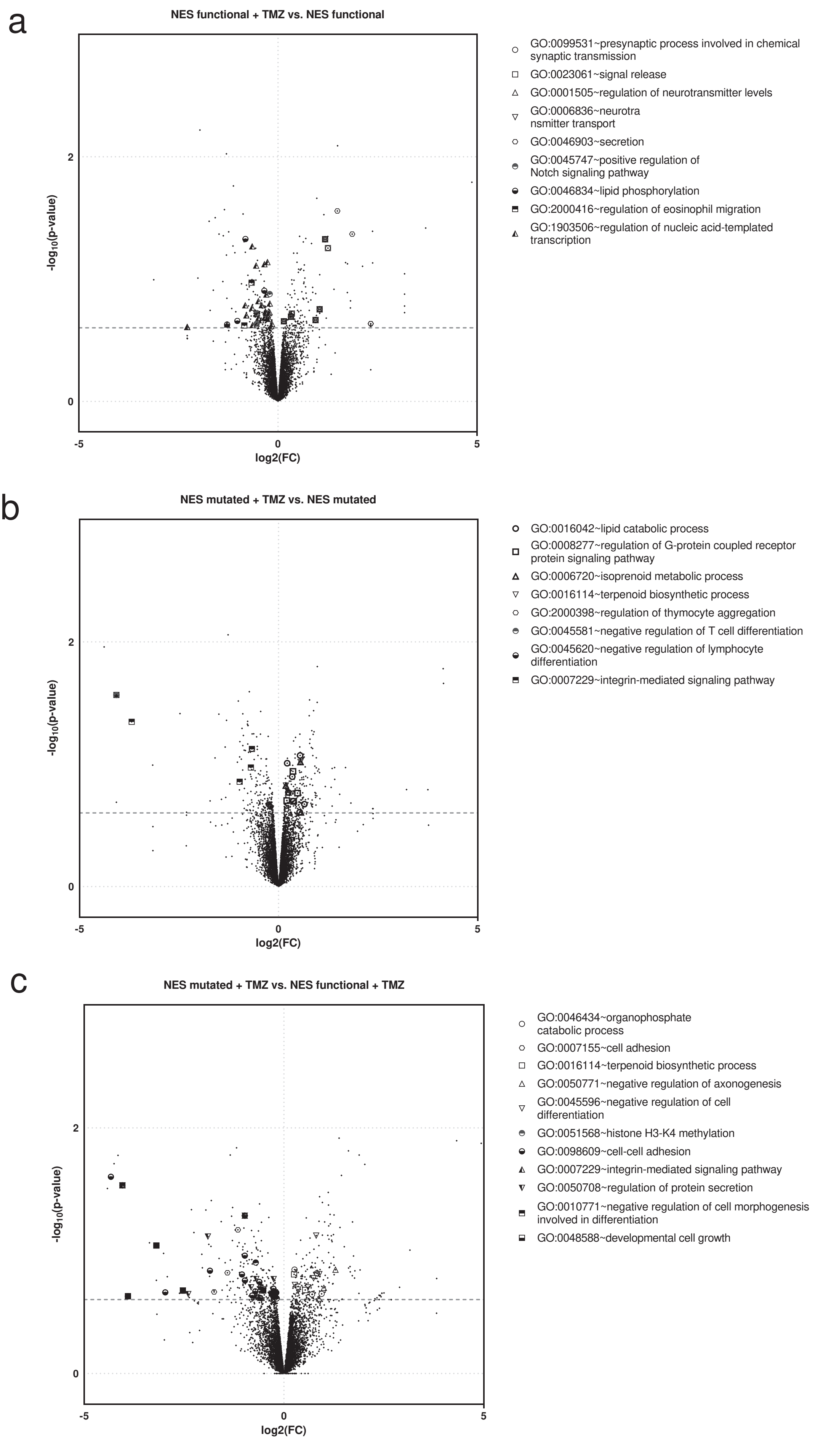

Suppl. Figure S8 a-c Volcano plots showing differentially expressed genes and associated biological processes of Survivin cell clones with functional or mutated nuclear export sequence (NES), exposed to TMZ and the normalized comparison of both clones under TMZ exposure. The dashed line indicates the p-value cutoff (-log10 (p) ≤ 0.60206), depicted GO-Terms and their identifiers are listed on the right. The data represent two independent biological replicates in technical duplicates each of the two different LN229 Survivin-expressing clones, the Surv-GFP-D6 versus SurvNESmut-L2

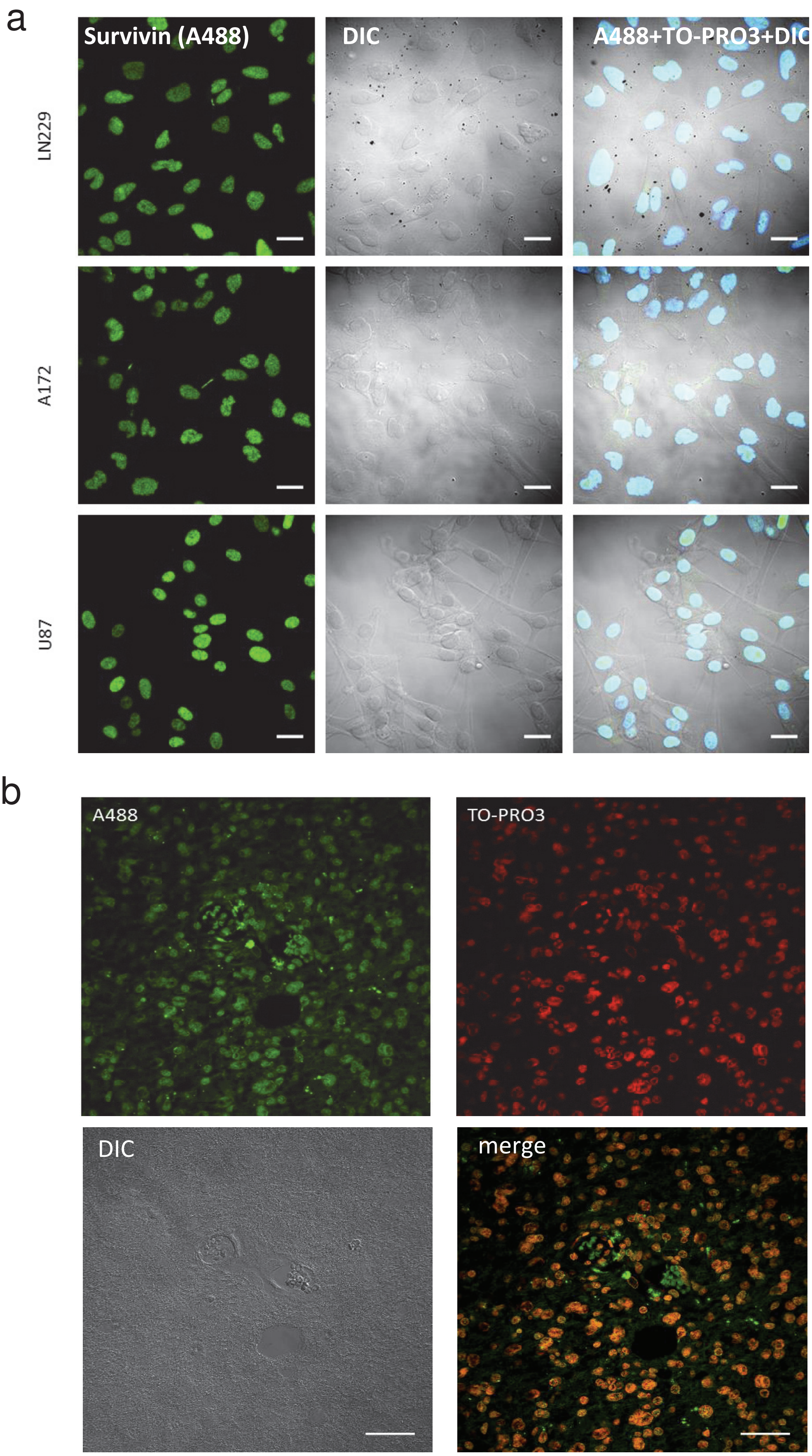

Suppl. Figure S9 a Immunofluorescence images of formaldehyde-fixed GB cell lines stained for Survivin (green, A488). Nuclei were stained with TO-PRO3 (blue) and differential interference contrast (DIC) images were overlaid (grayscale) to visualize cell structures. Scale bars equate 20 µm. **b** Immunofluorescence of a FFPE tumor section of an anaplastic oligodendroglioma (grade III) stained for Survivin (green, A488). Nuclear staining was performed with TO-PRO3 (shown in red). Differential interference contrast (DIC) images were overlaid to visualize cell structures (grayscale). Scale bars equate 50 µm

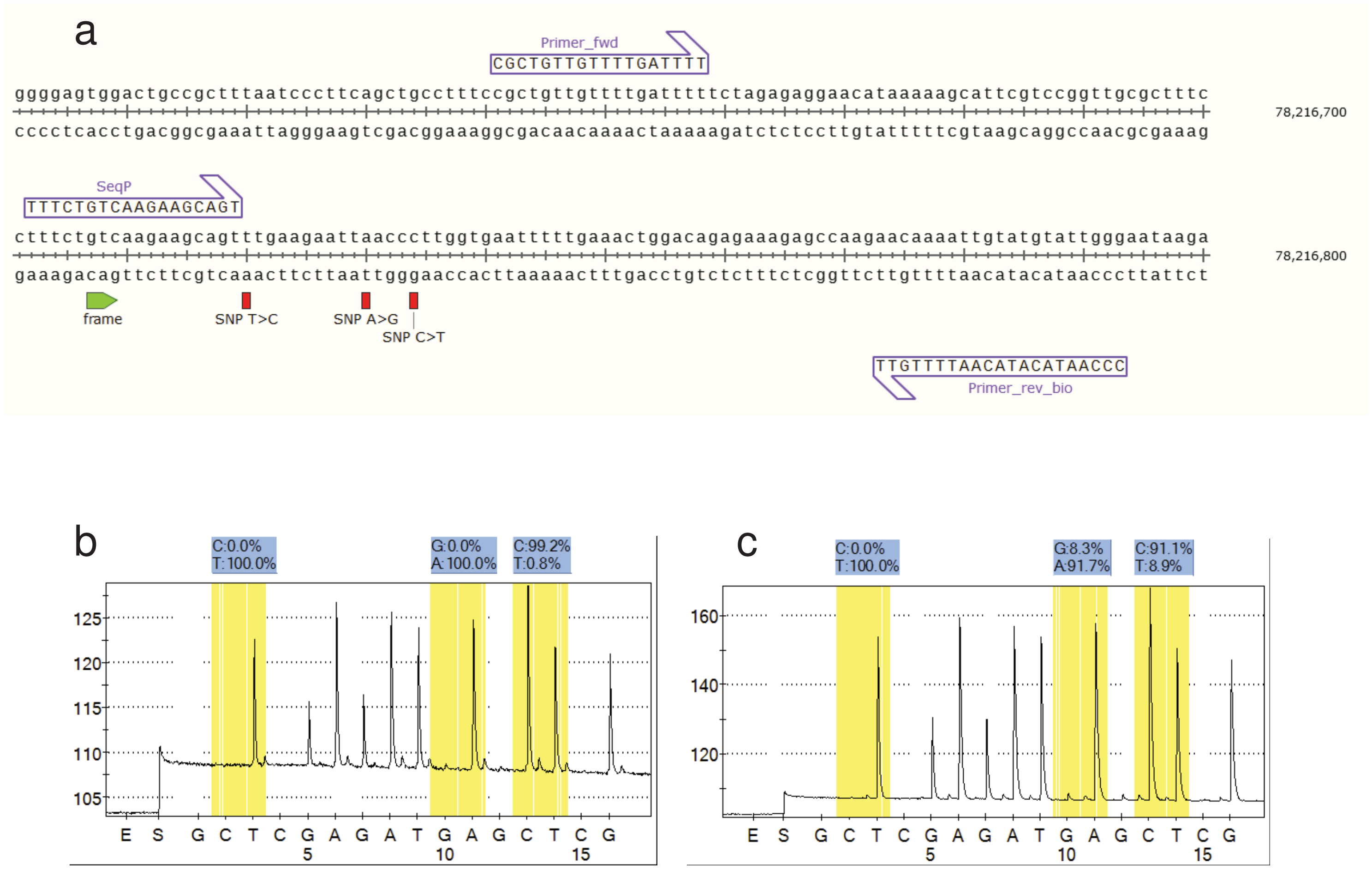

Suppl. Figure S10 a Setup of PCR and pyrosequencing primers, covering inactivating mutations in the NES of BIRC5. Two transition mutations at position 278 and 292 and one silent mutation at position 288 were analyzed in HGG samples. **b-c** Pyrosequencing results for detection of NES mutations in HGG patient samples. **b** Patient sample showing wild-type Survivin sequence and **c** Patient sample showing deviations from wild-type Survivin sequence

a

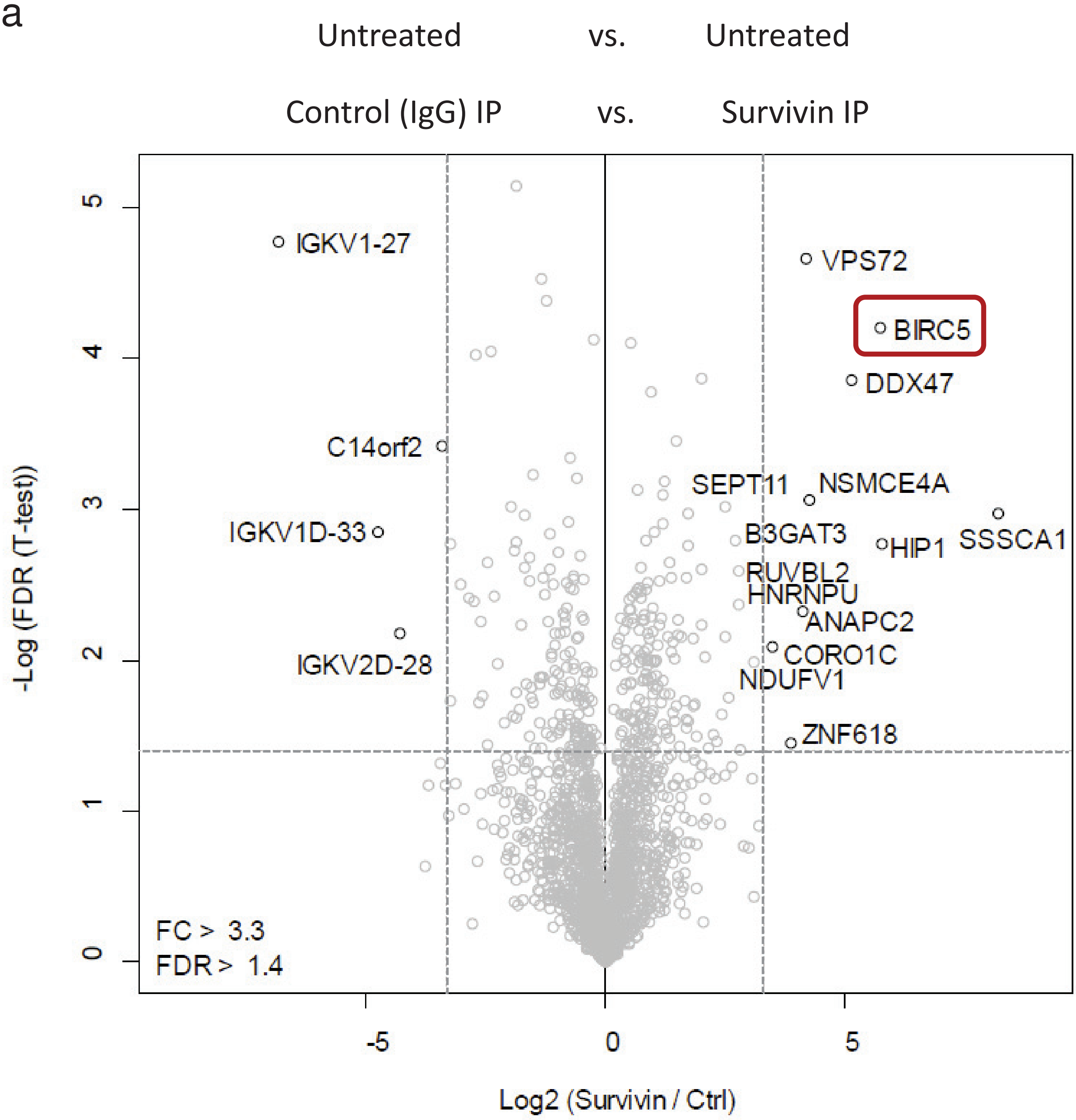

Suppl. Figure S11a Volcano plot analysis showing putative interactors of Survivin after co-immunoprecipitation of nuclear proteins and mass spectrometry-based interactomics in untreated LN229 cells

b

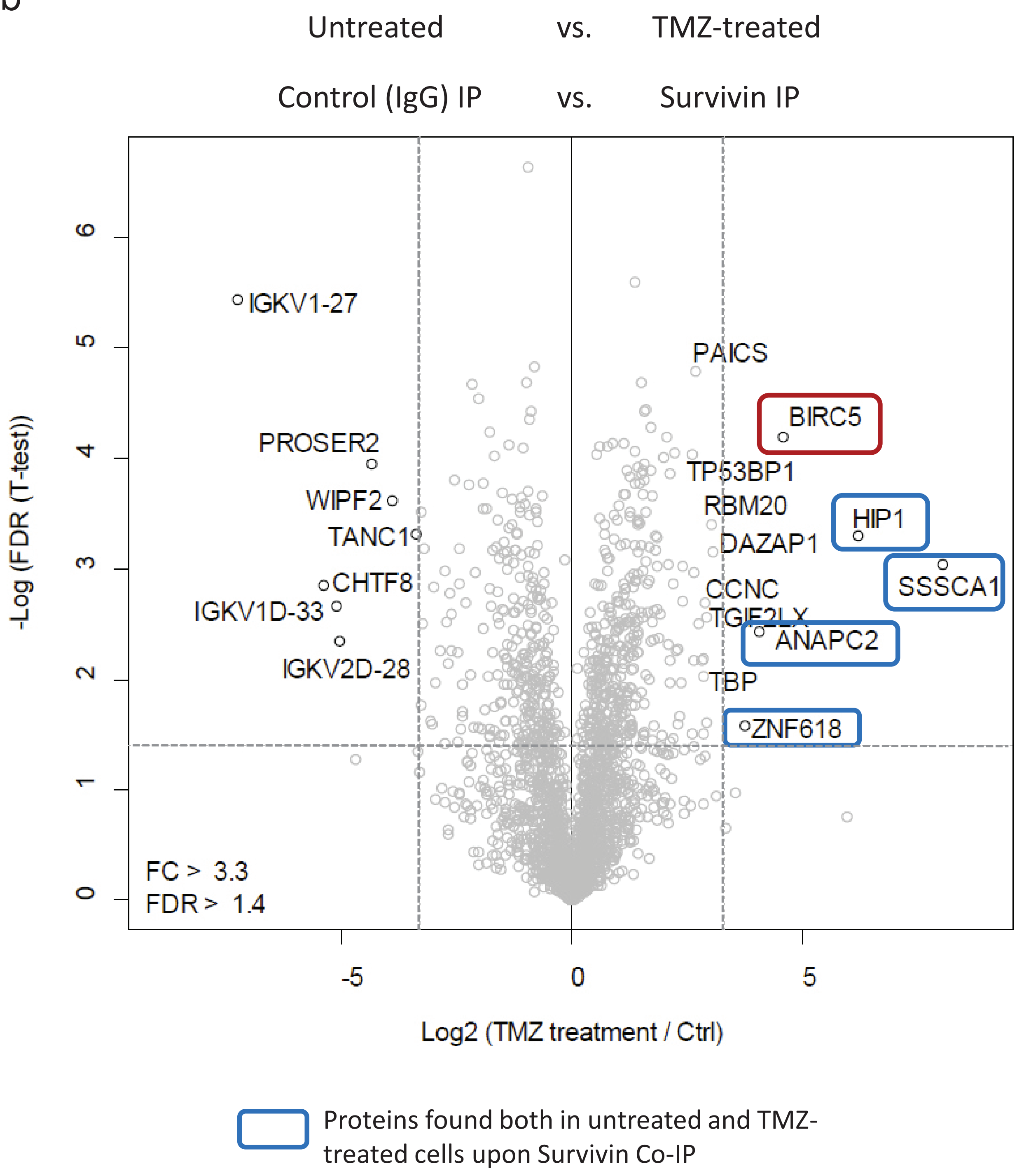

Suppl. Figure S11b Volcano plot analysis showing putative interactors of Survivin after co-immunoprecipitation of nuclear proteins and mass spectrometry-based interactomics in untreated versus TMZ-treated LN229 cells

c

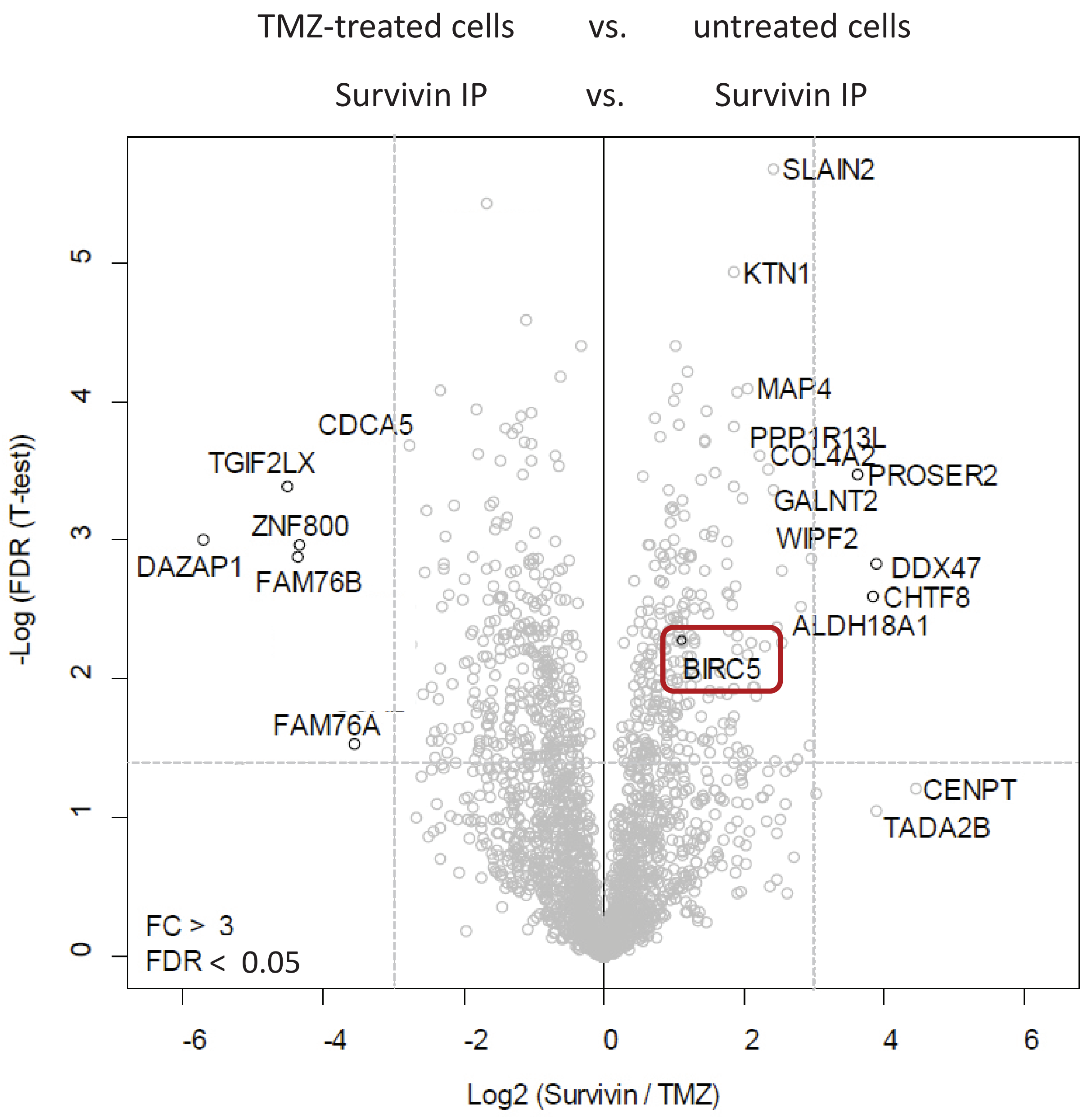

Suppl. Figure S11c Volcano plot analysis showing putative interactors of Survivin after co-immunoprecipitation of nuclear proteins and mass spectrometry-based interactomics in untreated versus TMZ-treated LN229 cells.

d

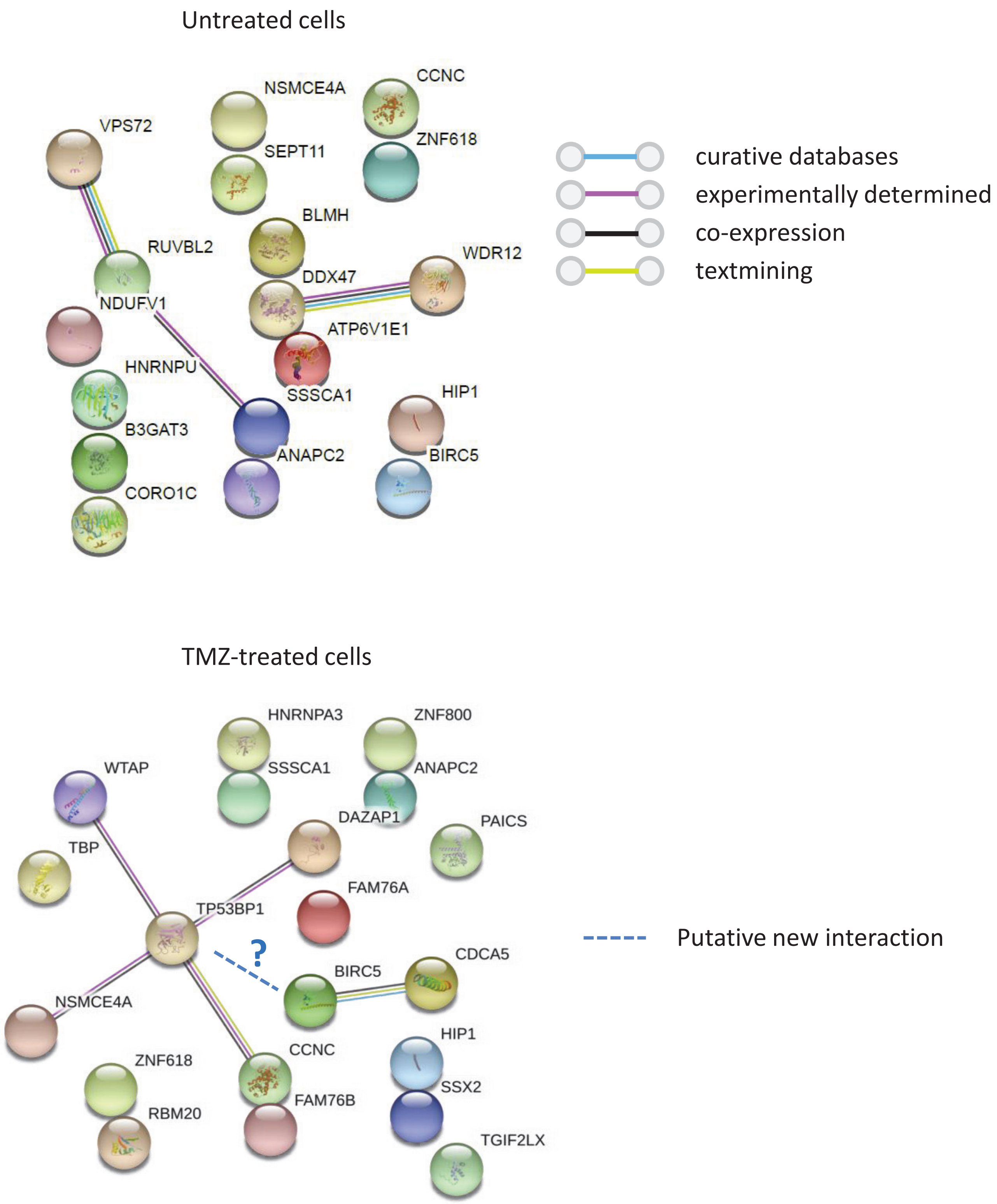

Suppl. Figure S11d: String network analysis showing reported and putative new interactions (pathways) based on the co-immunoprecipitated nuclear proteins and mass spectrometry-based interactomics in untreated *versus* TMZ-treated LN229 cells

Supplementary Material & Methods

DNA-dependent protein kinase (DNA-PK) assay

For quantification of the DNA-dependent protein kinase (DNA-PK) activity SignaTECT DNA-PK assay system (Promega) was used. This assay uses radioactively labeled [γ-^{32}P]-ATP (Perkin Elmer) for transferring ^{32}P to a p53-derived peptide substrate by DNA-PK. Due to the relatively short half-life of ^{32}P radioactivity had to be ordered separately for each assay performed. The biotinylated substrate is captured on'a streptavidin matrix (SAM2® Biotin Capture Membrane) and radioactivity is then measured in a liquid scintillation counter (Canberra Packard Central Europe). Two cell pellets were generated for each treatment/time-point. For analysis of the DNA-PK activity, nuclear extracts were prepared with lysis buffer 1. The cell pellet was resuspended in lysis buffer 1 with 0.5% NP-40, containing protease inhibitors. For cell lysis, the suspension was incubated on ice for 5 min and then centrifuged (5 min, 3200 rpm, 4 C). Supernatant was discarded.

To retain functionality of the DNA-PK catalytic subunit (CS) nuclei were resuspended in lysis buffer 1 (without NP-40) to get rid of remaining detergent. After centrifugation (5 min, 3200 rpm, 4 C), nuclei were resuspended in 100 ml extraction buffer (20 mM HEPES, 450 mM NaCl, 0.2 mM EDTA, 25%glycerol). Protease inhibitors were added freshly to the extraction buffer (0.5 mM DTT, 0.5 mM PMSF and 1 x Complete (Roche)). Samples were then subjected to 5 repeated freeze-thaw cycles between liquid nitrogen and 37°C. After centrifugation (5 min, 18407 g, 4°C), the cleared supernatant was then analyzed for total protein amount by Bradford protein assay. No DNA-PK activator (calf thymus DNA) was used for this experiment (refer to the manufacturers protocol), since the removal step of endogenous DNA by treatment with DEAE-sepharose was omitted. Thus, endogenous DNA was used as intrinsic activator of the DNA-PK enzyme, instead of an artificial activator of DNA-PK. An assay reaction mix was prepared as follows: 2.5 µL DNA-PK control buffer, 5 µL 5x reaction buffer, 2.5 µL DNA-PK biotynilated peptide substrate, 0.2 µL BSA (10 mg/mL), 5 µL γ-^{32}P-ATP mix (containing 4.5 µL 0.1 mM ATP + 0.5 µL [γ-^{32}P]-ATP). The reaction mix was pre-incubated at 30°C for 5 min. According to the enzyme sample with the lowest total protein concentration, remaining samples were adjusted to the same amount of protein in 9.75 µL maximum volume. Enzyme dilution buffer was prepared, using 1x reaction buffer with a final BSA concentration of 0.1 mg/mL. 9.75 µL enzyme sample were incubated with 15.2 µL Reaction mix for 5 min at 30°C. Upon termination with 12.5 µL termination buffer, samples were kept on ice before being spotted onto a prenumbered streptavidin membrane square (provided). Several washing steps of the membrane were performed to rinse off excess free [γ-^{32}P]-ATP and non-biotinylated proteins under repeated shaking: 1 x 30 s with 100 mL 2 M NaCl, 3 x 2 min with 100 mL 2 M NaCl, 4 x 2 min with 100 mL 2 M NaCl +1% H_3PO_4, 2 x 30 s with 100 mL deionized water. Radioactive waste was collected according to general regulations. After washing, the membrane was allowed to dry at RT for 30-60 min. Membrane squares were separated and transferred into vials for liquid scintillation, filled with 2 mL scintillation cocktail (Rotiszint, Carl Roth). Radiation in each sample was detected as counts per minute (CPM) on a liquid scintillation analyzer with 3 min counting time.

Mass spectrometry-based proteome analysis

Nuclear extract preparation and co-immunoprecipitation

The cells were left untreated or were treated with 50 µM TMZ, and 48 h later nuclear extracts were isolated using Nuclear Complex Co-IP Kit (Active Motif). Nuclear extracts were co-immunoprecipitate with the antibody against Survivin using magnetic beads (SureBeads™, Bio-Rad). The immunoprecipitate complex was eluted from the beads using LDS sample buffer (Life Technologies).

In-gel digestion of samples

Immunoprecipitated proteins were boiled in sample buffer supplemented with 1 mM DTT. Proteins were then alkylated by incubating them with 5.5 mM chloroacetamide for 30 minutes at room temperature. Proteins were resolved on 4-12% gradient SDS-PAGE gels (Life Technologies). The gels were stained using Colloidal Blue Staining Kit (Life Technologies) and digested in-gel using trypsin. The peptides were extracted from the gel pieces and desalted on C18 material (1).

MS analysis

Peptide fractions were analyzed on a quadrupole Orbitrap mass spectrometer (Q Exactive Plus, Thermo Scientific) equipped with a UHPLC system (EASY-nLC 1000, Thermo Scientific) as described (2, 3). Peptide samples were loaded onto C18 reversed phase columns (15 cm length, 75 µm inner diameter, 1.9 µm bead size) and eluted with a linear gradient from 8 to 40% acetonitrile containing 0.1% formic acid in 2-hours. The mass spectrometer was operated in data dependent mode, automatically switching between MS and MS^2 acquisition. Survey full scan MS spectra (m/z 300 – 1700) were acquired in the Orbitrap. The 10 most intense ions were sequentially isolated and fragmented by higher-energy C-trap dissociation (HCD) (4). An ion selection threshold of 5,000 was used. Peptides with unassigned charge states, as well as with charge states less than +2 were excluded from fragmentation. Fragment spectra were acquired in the Orbitrap mass analyzer.

Peptide identification

Raw data files were analyzed using MaxQuant (development version 1.5.2.8) (5). Parent ion and MS^2 spectra were searched against a database containing 88,473 human protein sequences obtained from the UniProtKB released in 2016 using Andromeda search engine (6). Spectra were searched with a mass tolerance of 6 ppm in MS mode, 20 ppm in HCD MS^2 mode, strict trypsin specificity and allowing up to 3 miscleavages. Cysteine carbamidomethylation was searched as a fixed modification, whereas protein N-terminal acetylation and methionine oxidation were searched as variable modifications. Match between runs and LFQ were selected. The data set was filtered based on posterior error probability (PEP) to arrive at a false discovery rate of below 1% estimated using a target-decoy approach (7).

Computational analysis

The 'proteingroups' data set was filtered for contaminants and reverse hits in the Perseus software (8). Statistical analysis for identifying interactors was performed using t-test on LFQ intensities as described (9). Functional protein interaction network analysis was performed using interaction data from the STRING database(10). Only interactions with a score > 0.7 are represented in the networks. Cytoscape version 3.7.2 was used for visualization of protein interaction networks (11).

References (Mass spectrometry-based proteome analysis)

1. Rappsilber,J., Ishihama,Y. and Mann,M. (2003) Stop and go extraction tips for matrix-assisted laser desorption/ionization, nanoelectrospray, and LC/MS sample pretreatment in proteomics. *Anal. Chem.*, **75**, 663–70.
2. Michalski,A., Damoc,E., Hauschild,J.P., Lange,O., Wieghaus,A., Makarov,A., Nagaraj,N., Cox,J., Mann,M. and Horning,S. (2011) Mass spectrometry-based proteomics using Q Exactive, a high-performance benchtop quadrupole Orbitrap mass spectrometer. *Mol Cell Proteomics*, **10**, M111 011015.
3. Kelstrup,C.D., Young,C., Lavallee,R., Nielsen,M.L. and Olsen,J. V (2012) Optimized fast and sensitive acquisition methods for shotgun proteomics on a quadrupole orbitrap mass spectrometer. *J Proteome Res*, **11**, 3487–3497.
4. Olsen,J. V, Macek,B., Lange,O., Makarov,A., Horning,S. and Mann,M. (2007) Higher-energy C-trap dissociation for peptide modification analysis. *Nat Methods*, **4**, 709–712.
5. Cox,J. and Mann,M. (2008) MaxQuant enables high peptide identification rates, individualized p.p.b.-range mass accuracies and proteome-wide protein quantification. *Nat. Biotechnol.*, **26**, 1367–1372.
6. Cox,J., Neuhauser,N., Michalski,A., Scheltema,R.A., Olsen,J. V and Mann,M. (2011) Andromeda: A peptide search engine integrated into the MaxQuant environment. *J. Proteome Res.*, **10**, 1794–1805.
7. Elias,J.E. and Gygi,S.P. (2007) Target-decoy search strategy for increased confidence in large-scale protein identifications by mass spectrometry. *Nat Methods*, **4**, 207–214.
8. Tyanova,S., Temu,T., Sinitcyn,P., Carlson,A., Hein,M.Y., Geiger,T., Mann,M. and Cox,J. (2016) The Perseus computational platform for comprehensive analysis of (prote)omics data. *Nat. Methods*, **13**, 731–740.
9. Hubner,N.C., Bird,A.W., Cox,J., Splettstoesser,B., Bandilla,P., Poser,I., Hyman,A. and Mann,M. (2010) Quantitative proteomics combined with BAC TransgeneOmics reveals in vivo protein interactions. *J. Cell Biol.*, **189**, 739–754.
10. Franceschini,A., Szklarczyk,D., Frankild,S., Kuhn,M., Simonovic,M., Roth,A., Lin,J., Minguez,P., Bork,P., Von Mering,C., *et al.* (2013) STRING v9.1: Protein-protein interaction networks, with increased coverage and integration. *Nucleic Acids Res.*, **41**, D808-15.
11. Saito,R., Smoot,M.E., Ono,K., Ruscheinski,J., Wang,P.L., Lotia,S., Pico,A.R., Bader,G.D. and Ideker,T. (2012) A travel guide to Cytoscape plugins. *Nat Methods*, **9**, 1069–1076.
